# Electrofusion Stimulation Is an Independent Factor of Chromosome Abnormality in Mice Oocytes Reconstructed *via* Spindle Transfer

**DOI:** 10.3389/fendo.2021.705837

**Published:** 2021-07-28

**Authors:** Wei Wang, Suxia Shao, Wei Chen, Weizhou Wang, Yunhai Chuai, Yunfei Li, Yiming Guo, Shujie Han, Mingming Shu, Qihang Wang, Lei Zhang, Wei Shang

**Affiliations:** ^1^Department of Histology and Embryology, Hebei Medical University, Shijiazhuang, China; ^2^Department of Obstetrics and Gynecology, The Sixth Medical Center of Chinese People’s Liberation Army (PLA) General Hospital, Beijing, China; ^3^Department of Obstetrics and Gynecology, Chinese PLA General Hospital, Beijing, China; ^4^Department of Reproductive Medicine, Harrison International Peace Hospital, Hengshui, China; ^5^Department of Biology, Kenneth P. Dietrich School of Art & Science, University of Pittsburgh, Pittsburgh, PA, United States; ^6^Navy Clinical Medical School, Anhui Medical University, Beijing, China; ^7^Department of Reproductive Medicine, First Hospital of Tsinghua University, Beijing, China

**Keywords:** spindle transfer, electrofusion, electrofusion stimulation, premature activation, chromosome abnormality

## Abstract

Oocytes reconstructed by spindle transfer (ST) are prone to chromosome abnormality, which is speculated to be caused by mechanical interference or premature activation, the mechanism is controversial. In this study, C57BL/6N oocytes were used as the model, and electrofusion ST was performed under normal conditions, Ca^2+^ free, and at room temperature, respectively. The effect of enucleation and electrofusion stimulation on MPF activity, spindle morphology, γ-tubulin localization and chromosome arrangement was compared. We found that electrofusion stimulation could induce premature chromosome separation and abnormal spindle morphology and assembly by decreasing the MPF activity, leading to premature activation, and thus resulting in chromosome abnormality in oocytes reconstructed *via* ST. Electrofusion stimulation was an independent factor of chromosome abnormality in oocytes reconstructed *via* ST, and was not related to enucleation, fusion status, temperature, or Ca^2+^. The electrofusion stimulation number should be minimized, with no more than 2 times being appropriate. As the electrofusion stimulation number increased, several typical abnormalities in chromosome arrangement and spindle assembly occurred. Although blastocyst culture could eliminate embryos with chromosomal abnormalities, it would significantly decrease the number of normal embryos and reduce the availability of embryos. The optimum operating condition for electrofusion ST was the 37°C group without Ca^2+^.

## Introduction

Spindle transfer (ST) is considered to be the most valuable therapeutic strategy for mitochondrial diseases and senile infertility, especially those with aging oocytes. Electrofusion ST has become the preferred method in mitochondrial replacement technology, because it doesn’t involve exogenous substances (1). Due to chromosome abnormalities in some of the reconstructed oocytes, the efficiency of ST technology is low. It is speculated that ST may cause mechanical interference in the spindle. Since the spindle along with chromosomes is not membrane-wrapped (2–9), enucleation or electrofusion stimulation may disrupt the function of the cytoskeleton, which may lead to abnormal chromosome segregation when the reconstructed oocyte is activated by subsequent fertilization (7, 8, 10, 11). It is also suspected that premature activation may lead to abnormal chromosome segregation (4), but this remains controversial.

At present, there are few researches on the effective inhibition of chromosome abnormality in oocytes reconstructed *via* spindle transfer. Daniel Paull (4) has suspected that temporary room temperature treatment is beneficial to maintain chromosome stability, which may make the spindle disappear temporarily to inhibit premature activation. But it remains unknown whether there is a correlation between the temporary disappearance of the spindle and inhibition of premature activation. Since oocytes are particularly sensitive to temperature, cooling treatment for more than 10 minutes may cause irreversible spindle damage (12–14), and change in incubation temperature as little as 0.5°C significantly affects mouse embryo development (15). In addition, a slight increase in incubation temperature may promote tubulin assembly, enhance the spindle birefringence, and make the spindle clearer under the microscope (16). With the increase in temperature or the extension of the high temperature, the spindle microtubules aggregation occurs, the spindle will also disappear but will not reappear after the temperature returns to normal, causing irreversible effects on oocytes (16–18). Besides, studies have shown that intracellular calcium oscillations triggered by sperm penetration during fertilization (19) and Ca^2+^ influx induced by mechanical or chemical operation (20) can both lead to decreased kinase activity, activation of oocytes and recovery of meiosis (21). Thus, some study has speculated that ST manipulation in a Ca^2+^ free medium may avoid spontaneous activation, but this has not been confirmed (3, 22).

In addition, MPF plays an important role in oocyte activation (23, 24). When oocytes are fertilized or parthenogenetically activated, the Ca^2+^ concentration increases instantaneously and cytostatic factor (CSF) expression decreases, resulting in the decrease or even disappearance of MPF activity and chromosome segregation, prompting oocytes to enter meiotic anaphase II (25). Moreover, premature activation in somatic cell nuclear transfer (SCNT) reconstructed embryos leads to abnormal spindles and chromosomes, as well as the expression of spindle related proteins (26, 27). γ-tubulin is an important regulatory protein involved in microtubule nucleation and spindle assembly that is located at the poles of the spindle in MII oocytes. If abnormal, γ-tubulin will dissociate from the poles, and become irregularly scattered in the spindle microtubulin or in the cytoplasm (24).

In this study, C57BL/6N oocytes were used as the model, and electrofusion ST was performed under normal condition, Ca^2+^ free, and at room temperature, respectively. The effects of enucleation and electrofusion stimulation on MPF activity, spindle morphology, γ-tubulin localization and chromosome arrangement were compared to verify the existence and occurrence of premature activation and to subsequently clarify the factors and mechanism for chromosome abnormality in mice oocytes reconstructed *via* ST, which would thus optimize ST technology and promote its clinical transformation.

## Materials and Methods

### Antibodies and Reagents

Monoclonal anti-β-tubulin-FITC (F2043-2ML) and DAPI (MBD0015-1ML) were purchased from Sigma-Aldrich. γ-Tubulin Monoclonal Antibody (4D11, MA1850) was obtained from Invitrogen. Goat Anti-Mouse IgG H&L (Dy Light^®^594, ab96881) was purchased from Abcam. A Mouse MPF Elisa Kit (DG94780Q-96T) was obtained from Dogesce, China. A BTXpress Cytofusion Medium C (47-0001) was obtained from BTX. A Gamate buffer (K-SIGB-20), Sperm medium (K-SISM-100), Fertilization medium (K-SIFM-20), Cleavage medium (K-SICM-20), and Blastocyst medium (K-SIBM-20) were purchased from Sydney IVF. G-PGD (10074) was obtained from Vitrolife. All other chemicals were purchased from Sigma-Aldrich, unless stated otherwise.

### Oocyte Retrieval and Culture

C57BL/6N mice (female, 6-8 weeks old; male, 7-8 months old) were purchased from Beijing Vital River Laboratory Animal Technology Co. Ltd. This study was reviewed and approved by the Institutional Animal Care and Use Committee of the Sixth Medical Center of China PLA General Hospital (HZKY-PJ-2019-3). The number of mice, oocytes and replications used in each group in this study were shown in **Supplemental Table S1**. Female mice were administered 10 IU of pregnant-mare serum gonadotropin (PMSG) and 48 h later 10 IU of human chorionic gonadotropin (HCG) (28). To avoid the effects of anesthesia, euthanasia was performed *via* cervical dislocation 14-16 hr after HCG injection, and the oviducts were isolated. Following the removal of cumulus cells with 40 IU hyaluronidase, MII oocytes were collected and incubated in a fertilization medium (FM) under liquid paraffin oil in a 37°C, 6% CO_2_, 5% O_2_ humidified incubator (29).

### Spindle Transfer

Oocytes were exposed to gamate buffer with 7.5μg/ml Cytochalasin B (CB) for 5 min at 37°C before manipulation. Then the dish was placed onto the warm stage of an Olympus IX71 inverted microscope equipped with micromanipulators. A slot was made in the zona pellucida, using the Saturn Active Laser System (RI, Saturn Active, 6-47-500, UK) with several pulses of 100-200μs. The spindle was then gently aspirated into the micromanipulation needle and transferred into the perivitelline space of an enucleated donor cytoplast. After that, the reconstructed oocytes were transferred into a CB free gamete buffer and incubated for 10 min in a 37°C humidified incubator, as shown in **Supplemental Figure S1**.

Notes: the 37°C treatment group and the 25°C treatment group represented that after enucleation ST reconstructed oocytes were treated at 37°C and 25°C for 5 min before electrofusion treatment, respectively.

### Electrofusion

Membrane fusion between the spindle and the donor cytoplast was initiated by placing it into BTXpress Cytofusion Medium C between gold electrodes (BEX, LF501G1, Japan). Different electrical pulse in each group listed in **Table 1** was delivered by an Electro Cell Fusion System (BEX, CFB16-HB, Japan) at room temperature. The reconstructed oocytes were then washed twice and transferred to FM for 20-30 min to check the fusion status.

**Table 1:** Different electrical pulse in each group.

Group	Pulse type	Voltage (kV/cm)	Pulse length (μs)	Pulse number
SEF	DC	1.50	150	1
DEF	DC	1.50	150	2
TEF	DC	1.50	150	3
MII-SEF	DC	1.50	150	1
MII-DEF	DC	1.50	150	2
MII-TEF	DC	1.50	150	3
2*MII-DEF1/2	DC	0.75	150	2*2
3*MII-DEF1/3	DC	0.50	150	2*3
4*MII-DEF1/3	DC	0.50	150	2*4
Others	DC	1.50	150	2

SEF, DEF, and TEF was the single electrofusion group, the double electrofusion group, and the triple electrofusion group, respectively. MII-SEF, MII-DEF and MII-TEF respectively represented the single electrofusion group, the double electrofusion group, the triple electrofusion group in MII oocytes. 2*MII-DEF1/2, 3*MII-DEF1/3 and 4*MII-DEF1/3 represented 1/2MII-DEF fusion voltage group with two consecutive shocks, 1/3MII-DEF fusion voltage group with three consecutive shocks and 1/3MII-DEF fusion voltage group with four consecutive shocks, respectively. Others represented the electrofusion parameters used in the other groups.

Notes: the Ca^2+^ group represented that the operating mediums such as gamete buffer used in spindle transfer process and Cytofusion Medium used in electrofusion process both contained Ca^2+^. The Ca^2+^ free group represented that the operating medium such as G-PGD used in spindle transfer and electrofusion process contained no Ca^2+^. In addition, the operating mediums used in spindle transfer and electrofusion process in other experiments were the same as those in the Ca^2+^ group.

### Fertilization and Culture

After a successful fusion, the reconstructed oocytes were transferred into FM to be co-incubated with sperm obtained from the cauda epididymis of C57BL/6N cultured in a sperm medium for 1h. 8 hours later, the zygotes were transferred into a cleavage medium for 2d, and then into a blastocyst medium for 2d, respectively.

### MPF Assay Procedure

The oocytes in each group were washed 3 times in a Ca^2+^ free PBS with 0.1% PVA, placed in tubes containing 15 μl of radio immunoprecipitation assay (RIPA) buffer containing a protease inhibitor cocktail tablet (Roche), vortexed on ice for 4–5 min, and then centrifuged at 4°C at 12,000 rpm for 15 min; The supernatant was collected and stored at –20°C until use. Assays of MPF level were performed using the Mouse MPF elisa kit (DOGESCE, China) following the manufacturer’s protocol.

### Immunofluorescence Staining

Immunofluorescence staining refers to the methods used by Zi-YunYi et al. (24), In brief, the oocytes in each group were fixed in 4% paraformaldehyde in a PBS with 0.5% Triton X-100 for 1 h at 4°C, followed by blocking in 3% BSA for 1 h at 37°C. Thereafter the oocytes were incubated with mouse monoclonal anti-γ-tubulin antibody (4D11, MA1850, invitrogen, 1:30) overnight at 4°C. After two washes (10 min each) in a washing buffer (0.1% PVA in PBS), the oocytes were labeled with Goat Anti-Mouse IgG H&L (DyLight^®^ 594, ab96881, Abcam, 1:30) for 1 h at 37°C. After two washes, the oocytes were stained with monoclonal anti-β-tubulin-FITC (F2043-2ML, Sigma, 1:30) for 1 h at 37°C, then co-stained with DAPI for 10 min at room temperature, followed by two more washes. Finally, the oocytes were mounted on glass slides with an antifading mounting medium (Sigma), and visualized with a confocal laser-scanning microscope (Nikon Ti2, Japan).

### Karyotype Analysis

Cultured for 4 days, blastocysts were washed in PBS for 2-3 times, then each blastocyst was transferred into a labeled centrifuge tube containing 2μl PBS. After brief centrifugation, the samples were immediately transferred to a refrigerator and stored at -80°C. Then, all samples were sent to genetic testing company (BASECARE, China) for karyotype analysis with sequencing depth of 1X.

### Statistical Methods

At least three replications were performed for each treatment and results obtained in different replications were pooled and analyzed together. The data was analyzed with SPSS23.0 statistical software, and GraphPad Prism 8.0 was used for plotting. Enumeration data such as oocyte/embryo proportions was expressed as a percentage (%), the comparison between groups was performed by the chi-square test, and measurement data such as the MPF activity was expressed as mean ± standard deviation, and was analyzed by univariate ANOVA. P<0.05 indicated statistical difference, P<0.01 indicated significant statistical difference, P<0.001 indicated extremely significant statistical difference, P>0.05 indicated no statistical difference.

## Results

### Electrofusion Stimulation Rather Than Enucleation, Was the Key Factor Causing Premature Activation in Mice ST Reconstructed Oocytes

**Figure 1:**
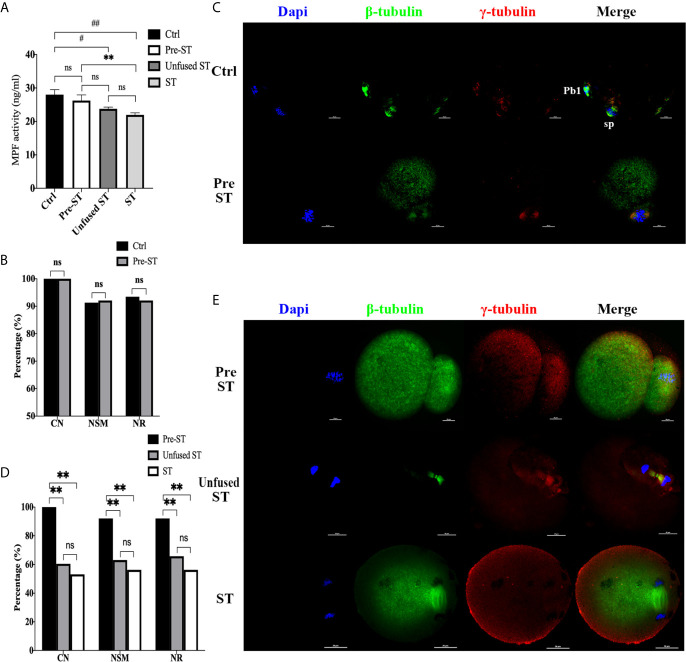
Electrofusion stimulation rather than enucleation might be the key factor causing premature activation in ST reconstructed oocytes. **(A)** After electrofusion stimulation, MPF activity was significantly reduced whether fused or not. (Ctrl, the control group, Pre-ST, the reconstructed oocytes before electrofusion group, Unfused ST, the unfused ST reconstructed oocytes group, ST, the fused ST reconstructed oocytes group). ^#^P<0.05, ^##^P<0.01, **P<0.01, ns P>0.05. Data is the MPF activity per 100 oocytes (ng/ml), expressed as mean ± standard deviation. **(B)** Enucleation did not cause abnormal chromosomes activity and spindle organization during meiosis. CN, chromosomal nondisjunction rate. NSM, normal spindle morphology rate. NR, normal γ-tubulin rate. ns P>0.05. Data is expressed as a percentage (%). Immunofluorescence staining results in **(C)** confirmed that the chromosome arrangement, spindle morphology, and γ-tubulin localization were normal in both groups. (Pb1, first polar body; sp, spindle). **(D)** Electrofusion stimulation, whether fused or not, would cause abnormal chromosomes activity and disrupted spindle organization during meiosis. Data is expressed as a percentage (%). **P<0.01, ns P>0.05. **(E)** Immunofluorescence staining. In Pre-ST, the chromosome arrangement, spindle morphology, and γ-tubulin location were all normal. In unfused ST, the chromosomes were separated to both poles of the spindle, with disordered arrangement. Spindle morphology was abnormal and γ-tubulin localization was disrupted. In the ST group, chromosomes were separated to both poles of the spindle. The spindle morphology was normal, while the γ-tubulin localization was disordered and scattered into the cytoplasm. DAPI labeled chromosomes (blue), anti-β-tubulin antibody coupled to FITC labeled spindles (green), and anti-γ-tubulin antibody coupled to DyLight 594 labeled γ-tubulin (red). Scale bar, 20μm.

To investigate whether and when premature activation occurred in the ST process, we first detected MPF activity in each procedure, including MII oocytes (Ctrl), reconstructed oocytes before electrofusion (pre-ST), unfused ST reconstructed oocytes (Unfused ST) and fused ST reconstructed oocytes (ST), with the results shown in **Figure 1A**. There was no significant difference in MPF activity between the Ctrl and pre-ST groups (P=0.3421). Compared with Ctrl, the MPF activity was significantly decreased in Unfused ST (P=0.0107) and ST (P=0.0012), and the decline was most significant in ST. Meanwhile MPF activity in ST was also significantly lower than that in pre-ST (P=0.0097), indicating that electrofusion stimulation significantly reduced the MPF activity in ST reconstructed oocytes.

Additionally, the effect of enucleation and electrofusion stimulation on the chromosome, spindle morphology, and γ-tubulin were compared, as shown in **Figures 1B–E**. In the Ctrl and Pre-ST group, chromosomes of oocytes were all arranged in the center of the spindle, and the spindle was normal, forming a typical bipolar, symmetrical, and spindle-shaped structure (P=0.895). γ-tubulin was located on both poles of the spindle (P=0.808). Conversely, the chromosomal nondisjunction rate (CN), normal spindle morphology rate (NSM), normal γ-tubulin rate (NR) in Unfused ST and ST were all significantly lower than those in Pre-ST (P<0.01, respectively). There was no significant difference in CN (P=0.533), NSM (P=0.557), and NR (P=0.414) between Unfused ST and ST. Electrofusion stimulation, whether fused or not, rather than enucleation, caused reduced MPF activity, abnormal chromosomes activity and disrupted spindle organization during meiosis, indicating that after electrofusion stimulation, premature activation occurred in ST reconstructed oocytes.

### The Electrofusion Stimulation Number in the ST Process Should be Minimized, No More Than 2 Times is Appropriate

**Figure 2:**
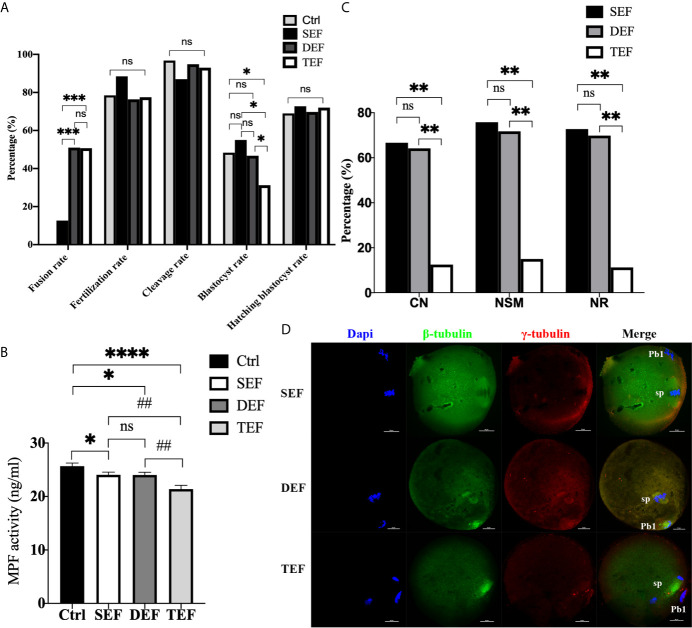
The electrofusion stimulation number in the ST process should be minimized, with no more than 2 times being appropriate. **(A)** Culture results. The fusion rate in DEF and TEF was significantly higher than that in SEF. The blastocyst rate in TEF decreased significantly, with no significant difference between Ctrl, SEF and DEF. SEF, DEF, and TEF are the single electrofusion group, the double electrofusion group, and the triple electrofusion group, respectively. The data is expressed as a percentage (%). *P<0.05, ***P<0.001, and ns P>0.05. **(B)** Single or double electrofusion had little effect on the MPF activity, while triple electrofusion resulted in a very significant decrease. The data is expressed as mean ± standard deviation. *P<0.05, ****P<0.0001. ^##^P<0.01, ns, P>0.05. Value, the MPF activity per 100 oocytes (ng/ml). **(C)** Immunofluorescence staining results indicated that CN, NSM, and NR in TEF were significantly lower than that in SEF and DEF, with no statistical difference between SEF and DEF. The data is expressed as a percentage (%). **P<0.01, ns P>0.05. **(D)** Representative images in SEF and DEF were both normal in terms of chromosomes, spindle morphology, and γ-tubulin location. In the TEF group, the chromosomes were separated in advance, the spindle morphology was abnormal, and γ-tubulin localization was disordered. Scale bar, 20μm.

To further define the inducement of ST premature activation, electrofusion was subdivided into 3 groups: the single electrofusion group (SEF), the double electrofusion group (DEF), and the triple electrofusion group (TEF). Culture results in **Figure 2A** showed that the fusion rate in DEF and TEF was significantly higher than that in SEF (P_DEF/SEF_<0.001, P_TEF/SEF_<0.001), with no statistical difference between DEF and TEF (P=0.945). Compared with Ctrl (P=0.040), SEF (P=0.048) and DEF (P=0.038), the blastocyst rate in TEF decreased significantly, and there was no significant difference in the blastocyst rate between Ctrl, SEF and DEF (P_Ctrl/SEF_=0.606, P_Ctrl/DEF_=0.847, P_SEF/DEF_=0.503). No significant difference existed in the fertilization rate (P_Ctrl/SEF_=0.261, P_Ctrl/DEF_=0.727, P_Ctrl/TEF_=0.869, P_DEF/SEF_=0.172, P_TEF/SEF_=0.211, P_DEF/TEF_=0.841), cleavage rate (P_Ctrl/SEF_=0.087, P_Ctrl/DEF_=0.563, P_Ctrl/TEF_=0.319, P_DEF/SEF_=0.173, P_TEF/SEF_=0.348, P_DEF/TEF_=0.605), and blastocyst hatching rate (P_Ctrl/SEF_=0.817, P_Ctrl/DEF_=0.942, P_Ctrl/TEF_=0.808, P_DEF/SEF_=0.848, P_TEF/SEF_=0.964, P_DEF/TEF_=0.846) between the 4 groups. Thus, increasing the electrofusion stimulation number could promote the fusion of ST reconstructed oocytes, but repeated electrofusion stimulation (≥3) might cause damage, and thus affect the developmental potential.

Furthermore, in **Figure 2B**, the MPF activity in SEF (P=0.0350) and DEF (P=0.0326) was statistically lower than that in Ctrl, with no statistical difference between SEF and DEF (P>0.05). Compared with SEF (P=0.0020), DEF (P=0.0021) and Ctrl (P<0.0001), MPF activity in TEF was the lowest one. The immunofluorescence staining shown in **Figures 2C, D** indicated that CN (P_TEF/SEF_<0.01, P_DEF/TEF_<0.01, P_DEF/SEF_=0.812), NSM (P_TEF/SEF_<0.01, P_DEF/TEF_<0.01, P_DEF/SEF_=0.679) and NR (P_TEF/SEF_<0.01, P_DEF/TEF_<0.01, P_DEF/SEF_=0.772) in SEF and DEF were all significantly higher than that in TEF, with no statistical difference between the two groups. We concluded that single or double electrofusion had little effect on mice oocytes reconstructed *via* ST, while triple electrofusion might have a negative effect, and that a threshold might exist in MPF inactivation.

**Figure 3:**
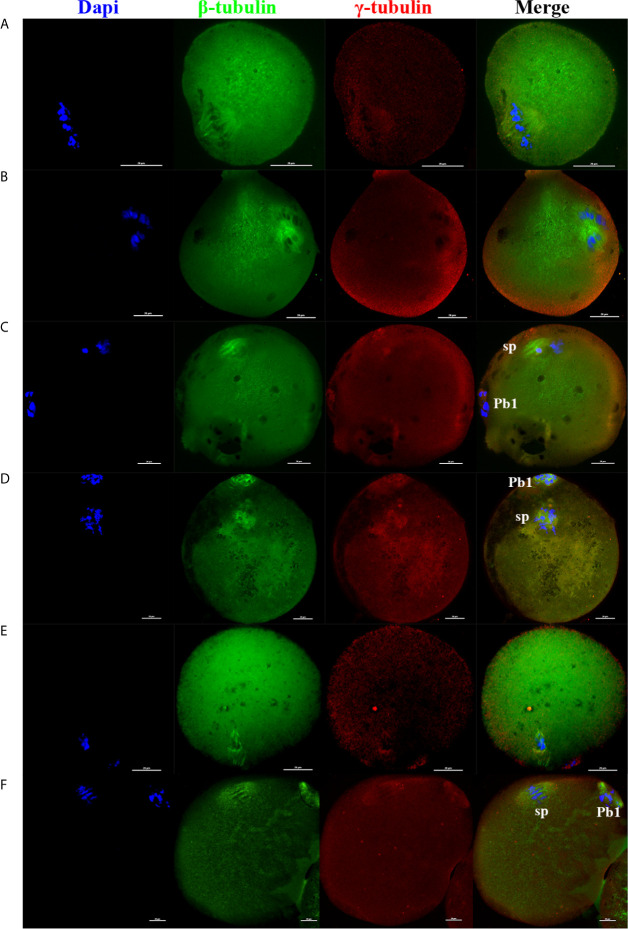
As the electrofusion stimulation number increased, several typical abnormalities in the chromosome arrangement and spindle assembly were generated, especially in TEF. Representative images from the immunofluorescence staining are shown. **(A)** The spindle was significantly wider, while the chromosomes and γ-tubulin localization were normal. **(B)** Chromosomes were multilaterally arranged outside the spindle, and microtubules were disordered, with disordered γ-tubulin localization, forming a multipolar spindle. **(C)** Chromosome distribution was disordered, some in the center of the spindle, others at one pole of the spindle, microtubules at one pole of the spindle were fractured, with the γ-tubulin dissociated from the poles of the spindle, irregularly scattered in the spindle microtubules or in the cytoplasm, forming a unipolar spindle; **(D)** Chromosomes had a disorderly arrangement throughout the whole spindle region. The spindle microtubules were fractured and disordered, with γ-tubulin dissociated from the poles of the spindle and dispersed irregularly on the microtubules, forming a poleless spindle. **(E)** The arrangement of the microtubules was disrupted, with the chromosomes having a disorderly arrangement in the center of the spindle. **(F)** The spindle was over-elongated. Scale bar, 20μm.

In addition, as the electrofusion stimulation number increased, several typical abnormalities in chromosome arrangement and spindle assembly occurred, especially in the TEF shown in **Figure 3**. Chromosome abnormality mainly included misaligned chromosomes in the metaphase-plate region of the spindle, disrupted chromosomes spread throughout the whole spindle region, with others at the pole of the spindle. There were several types of aberrant spindle organization, including fractured spindle microtubules, disordered arrangement, broadened spindles, over-elongated spindles, the absence of spindles, and abnormal spindle poles, including spindles with no poles, monopoles, and multipoles, etc. The localization of γ-tubulin, an important regulator of spindle organization at the spindle poles, was also disrupted, with γ-tubulin dissociated from the poles of the spindle, irregularly scattered in the spindle microtubules, or in the cytoplasm.

**Figure 4:**
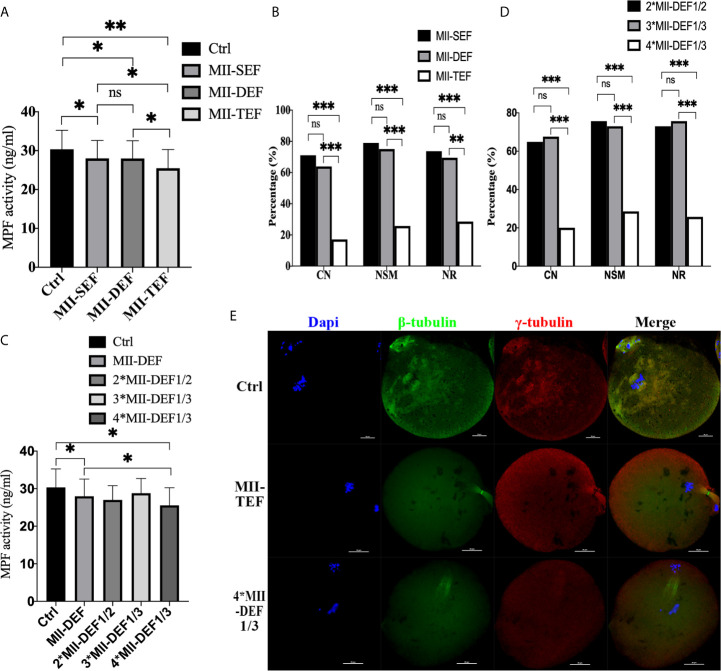
Electrofusion stimulation also caused premature activation in MII oocytes, which was significantly increased in both MII-TEF and 4*MII-DEF1/3, and both had a negative effect on chromosome arrangement and spindle assembly. **(A)** MPF activity. MII-SEF, MII-DEF and MII-TEF respectively represented the single electrofusion group, the double electrofusion group, the triple electrofusion group in MII oocytes. Data is expressed as mean ± standard deviation. *P<0.05, **P<0.01, ns, P>0.05. Value, the MPF activity per 100 oocytes (ng/ml). **(B)** CN, NSM, and NR in MII-TEF were significantly decreased. ***P<0.001, **P<0.01, ns, P>0.05. MPF activity in 4*MII-DEF1/3 was decreased in **(C)**, as well as CN, NSM, and NR in **(D)**. *P<0.05, ***P<0.001, ns P>0.05. **(E)** Representative images in Ctrl were normal in terms of chromosomes, spindle morphology, and γ-tubulin location. In MII-TEF and 4*MII-DEF1/3, the chromosomes were separated in advance, spindle morphology was abnormal, and the γ-tubulin localization was disordered. Scale bar, 20μm.

To further clarify whether premature activation was related to fusion state, we conducted direct electrofusion stimulation of MII oocytes (**Figures 4A, B, E**), which demonstrated that MPF activity in MII-SEF (P=0.0176) and MII-DEF (P=0.0194) was significantly lower than that in Ctrl, and there was no statistical difference between the two groups (P=0.7683), while the MPF activity in MII-TEF was the lowest one, compared with MII-SEF (P=0.0323), MII-DEF (P=0.0294) and Ctrl (P=0.0069). Immunofluorescence staining also confirmed that CN (P_MII-TEF/SEF_<0.001, P_MII-DEF/TEF_<0.001, P_MII-DEF/SEF_=0.511), NSM (P_MII-TEF/SEF_<0.001, P_MII-DEF/TEF_<0.001, P_MII-DEF/SEF_=0.687), and NR(P_MII-TEF/SEF_<0.001, P_MII-DEF/TEF_=0.001, P_MII-DEF/SEF_=0.686) in MII-TEF significantly decreased among the three group. MII-SEF, MII-DEF and MII-TEF respectively represented the single electrofusion group, the double electrofusion group, the triple electrofusion group in MII oocytes. We also found that repeated low intensity electrofusion stimulation (as shown in **Figures 4C–E**) also induced premature chromosome separation and abnormal spindle morphology and assembly, leading to premature activation. Compared with Ctrl, MPF activity in both MII-DEF (P=0.0237) and 4*MII-DEF1/3 (P=0.0111) was decreased, and that in 4*MII-DEF1/3 was significantly lower (P=0.0349). Meanwhile CN (P_4*MII-DEF1/3/2*MII-DEF1/2_<0.001, P_4*MII-DEF1/3/3*MII-DEF1/3_<0.001, P_2*MII-DEF1/2/3*MII-DEF1/3_ = 0.806), NSM (P_4*MII-DEF1/3/2*MII-DEF1/2_<0.001, P_4*MII-DEF1/3/3*MII-DEF1/3_<0.001, P_2*MII-DEF1/2/3*MII-DEF1/3_ = 0.790), and NR (P_4*MII-DEF1/3/2*MII-DEF1/2_<0.001, P_4*MII-DEF1/3/3*MII-DEF1/3_<0.001, P_2*MII-DEF1/2/3*MII-DEF1/3_ = 0.790) in 4*MII-DEF1/3 were all significantly reduced. 2*MII-DEF1/2, 3*MII-DEF1/3 and 4*MII-DEF1/3 represented 1/2MII-DEF fusion voltage group with two consecutive shocks, 1/3MII-DEF fusion voltage group with three consecutive shocks and 1/3MII-DEF fusion voltage group with four consecutive shocks, respectively. It could be seen that electrofusion stimulation also caused premature activation in MII oocytes, which was significantly increased in both MII-TEF and 4*MII-DEF1/3, with both having a negative effect on chromosome arrangement and spindle assembly.

**Figure 5:**
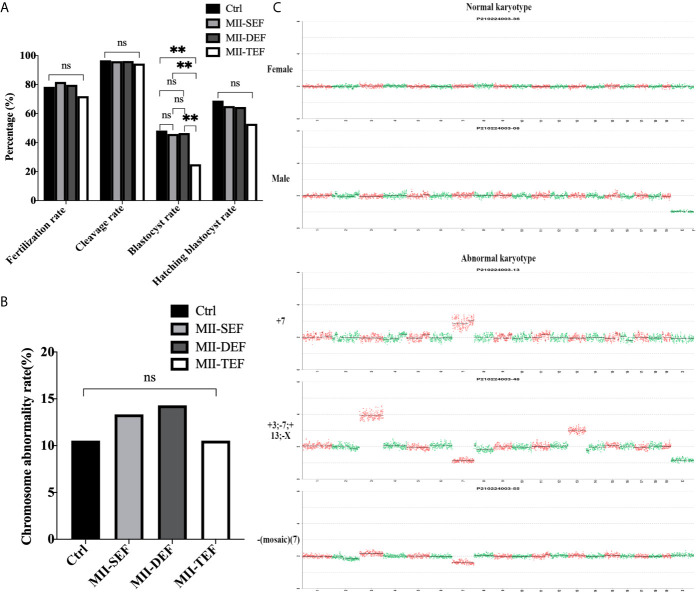
Effect of electrofusion stimulation number on the developmental potential and chromosome karyotype of non-ST embryos. **(A)** The blastocyst rate in MII-TEF was significantly reduced. No significant difference existed in the fertilization rate, cleavage rate, and blastocyst hatching rate among the 4 groups. Data is expressed as a percentage (%). **P<0.01, ns P>0.05. **(B)** There was no statistical difference in the chromosome abnormality rate in blastocysts among the 4 groups. ns P>0.05. **(C) **The upper two images were normal karyotypes of female and male mouse blastocysts, respectively, the lower three images were abnormal karyotypes, such as +7, +3; -7; +13; -X and -(Mosaic) (7).

Embryos in the Ctrl, MII-SEF, MII-DEF, and MII-TEF groups were cultured for 4 days. **Figure 5A** showed that the blastocyst rate in the MII-TEF group was significantly reduced (P_MII-TEF/Ctrl_=0.006, P_MII-TEF/SEF_=0.006, P_MII-TEF/DEF_=0.004), while there was no statistical difference between Ctrl, MII-SEF and MII-DEF (P_MII-Ctrl/SEF_=0.775, P_MII-Ctrl/DEF_=0.831, P_MII-SEF/DEF_=0.931). No significant difference existed in the fertilization rate (P_MII-TEF/Ctrl_=0.321, P_MII-TEF/SEF_=0.076, P_MII-TEF/DEF_=0.161, P_MII-Ctrl/SEF_=0.548, P_MII-Ctrl/DEF_=0.811, P_MII-SEF/DEF_=0.676), cleavage rate (P_MII-TEF/Ctrl_=0.516, P_MII-TEF/SEF_=0.592, P_MII-TEF/DEF_=0.564, P_MII-Ctrl/SEF_=0.836, P_MII-Ctrl/DEF_=0.862, P_MII-SEF/DEF_=0.967), and blastocyst hatching rate(P_MII-TEF/Ctrl_=0.277, P_MII-TEF/SEF_=0.373, P_MII-TEF/DEF_=0.397, P_MII-Ctrl/SEF_=0.737, P_MII-Ctrl/DEF_=0.694, P_MII-SEF/DEF_=0.949) among the 4 groups. However, a karyotype analysis (**Figures 5B, C**, **Supplemental Table S2** and **Figure S2**) showed that there was no statistical difference in the chromosome abnormality rate in blastocysts among the 4 groups (P_MII-TEF/Ctrl_=1.000, P_MII-TEF/SEF_=0.801, P_MII-TEF/DEF_=0.744, P_MII-Ctrl/SEF_=0.801, P_MII-Ctrl/DEF_=0.744, P_MII-SEF/DEF_=0.941). It could be seen that the blastocyst culture could eliminate embryos with chromosomal abnormalities in the MII-TEF group, so it would significantly decrease the number of normal embryos and reduce the availability of embryos.

### Transient Room Temperature Treatment After Enucleation Did Not Inhibit Premature Activation

**Figure 6:**
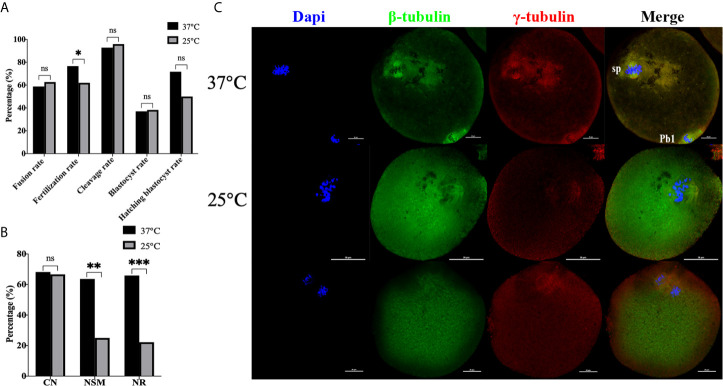
Transient room temperature treatment after enucleation did not inhibit premature activation. **(A)** Transient room temperature treatment did not significantly affect the development potential of ST reconstructed embryos, but had an adverse effect on fertilization. ns P>0.05, *P<0.05. **(B)** Immunofluorescence staining results showed that transient room temperature treatment could lead to abnormal spindle function in ST reconstructed oocytes. **P<0.01, ***P<0.001. Representative images are shown in **(C)**. For 37°C the images are normal. The middle 25°C showed that after transient room temperature treatment, the chromosomes were disordered, irregularly localized at the equatorial plate, with abnormal spindle morphology and γ-tubulin localization, while in the bottom 25°C images, the chromosomes separated in advance, the spindle microtubulin disappeared, and the γ-tubulin aggregated towards the middle of the spindle. 37°C and 25°C respectively represent the 37°C treatment group and the 25°C treatment group. Data is expressed as a percentage (%). Scale bar, 20μm.

Next, the effect of temperature (37°C, 25°C) on mice oocytes reconstructed *via* ST was compared, with the results shown in **Figure 6**. Culture results showed that transient room temperature treatment after enucleation had an adverse effect on fertilization (**Figure 6A**). There was no statistical difference in fusion rate (P=0.442), cleavage rate (P=0.446), blastocyst rate (P=0.879), and hatching blastocyst rate (P=0.093) between the two groups. The fertilization rate at 37°C (76.62% *vs* 62.03%, P=0.014) was significantly higher. Moreover, the immunofluorescence staining results (**Figures 6B, C**) indicated that no statistical difference existed between the two groups in terms of CN (P=0.886). NSM (P=0.001) and NR (P<0.001) at 37°C were both significantly higher than those at 25°C, indicating that transient room temperature treatment after enucleation did not inhibit premature activation, and might affect spindle function in ST reconstructed oocytes, reducing the fertilization rate in ST reconstructed oocytes.

### A Ca^2+^ Free Manipulation Medium Did Not Inhibit Premature Activation

**Figure 7:**
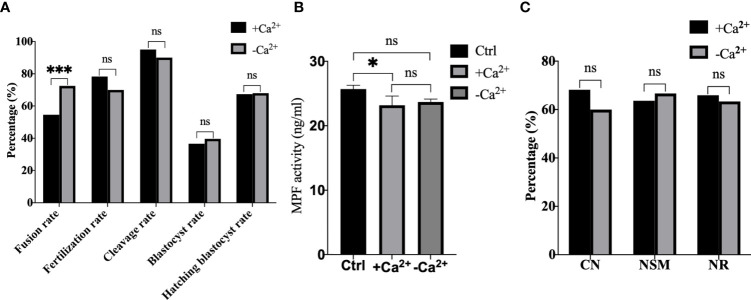
A Ca^2+^ free manipulation medium did not inhibit premature activation. **(A)** A Ca^2+^ free medium significantly improved the fusion rate of mouse reconstructed oocytes. Data is expressed as a percentage (%). ns P>0.05, ***P<0.001. **(B)** The MPF activity decreased significantly in +Ca^2+^, with no statistical difference between -Ca^2+^ and +Ca^2+^/Ctrl. Data is expressed as mean ± standard deviation. ns P>0.05, *P<0.05. Value, the MPF activity per 100 oocytes (ng/ml). **(C)** There was no statistical difference in CN, NSM, and NR between +Ca^2+^ and -Ca^2+^. ns P>0.05. Data is expressed as a percentage (%). +Ca^2+^ represents the Ca^2+^ medium group, and -Ca^2+^ represents the Ca^2+^ free medium group.

Afterwards, we performed electrofusion ST in a Ca^2+^ free medium to explore whether or not premature activation was inhibited, and the results were shown in **Figure 7**. Interestingly, the fusion rate of the Ca^2+^ free group (P<0.001) was extremely significantly higher, while there was no statistical difference in the fertilization rate (P=0.121), cleavage rate (P=0.166), blastocyst rate (P=0.674), and the hatching blastocyst rate (P=0.955) in **Figure 7A**. Moreover, the MPF activity, as shown in **Figure 7B**, decreased significantly in the Ca^2+^ group (P=0.0211), with no statistical difference between the Ca^2+^ group and Ca^2+^ free group (P=0.8060), or between the Ctrl group and Ca^2+^ free group (P=0.1405). Additionally, no statistical difference existed in terms of CN (P=0.469), NSM (P=0.789), and NR (P=0.820) between the Ca^2+^ group and Ca^2+^ free group (**Figure 7C**), indicating that a Ca^2+^ free manipulation medium did not inhibit premature activation, and that extracellular Ca^2+^ might not be the key factor causing calcium oscillations in ST reconstructed oocyte activation.

### The Optimum Operating Conditions for Electrofusion ST Technology Was the 37°C Group Without Ca^2+^

**Figure 8:**
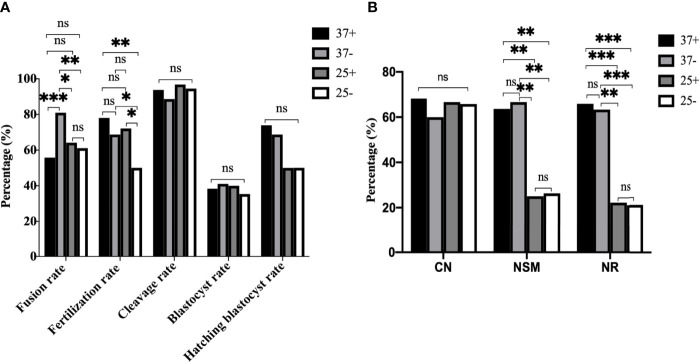
The optimum operating condition for electrofusion ST technology was the 37°C group without Ca^2+^. **(A)** Culture results showed that the fusion rate was the highest in 37-, the fertilization rate in 25- was the lowest, with no difference in the fertilization rate existing between 37+, 37-, and 25+. *P<0.05, **P<0.01, ***P <0.001, and ns P>0.05. **(B)** 25°C treatment after enucleation had adverse effects on the spindle morphology and γ-tubulin localization in mice oocytes reconstructed *via* ST. NSM and NR in 37+ and 37- were both significantly higher than those in 25+ and 25-, with no difference existing between 37+ and 37-. **P<0.01, ***P <0.001, and ns P>0.05. 37+, 37-, 25+, and 25- respectively represent the 37°C group containing Ca^2+^, the 37°C group without Ca^2+^, the 25°C group containing Ca^2+^, and the 25°C group without Ca^2+^. Data is expressed as a percentage (%).

To further optimize ST technology, we conducted a cross experiment as shown in **Figure 8**. In **Figure 8A** it can be seen that the fusion rate was the highest in 37-, followed by 25+, with no statistical difference between 37+ and 25- (P_37-/37+_<0.001, P_37-/25+_=0.022, P_37-/25-_=0.009, P_37+/25-_=0.492, P_37+/25+_=0.248, P_25+/25-_=0.714). The fertilization rate in 25- was the lowest, there was no difference between 37+, 37- and 25+ (P_37+/25-_=0.002, P_37-/25-_=0.047, P_25+/25-_=0.044, P_37-/37+_=0.204, P_37-/25+_=0.711, P_37+/25+_=0.459). No statistical difference existed among the four groups in the cleavage rate (P_37+/37-_=0.345, P_37+/25+_=0.536, P_37+/25-_=0.913, P_37-/25+_=0.201, P_37-/25-_=0.483, P_25+/25-_=0.691), blastocyst rate (P_37+/37-_=0.789, P_37+/25+_=0.878, P_37+/25-_=0.819, P_37-/25+_=0.931, P_37-/25-_=0.686, P_25+/25-_=0.750), and blastocyst hatching rate (P_37+/37-_=0.725, P_37+/25+_=0.157, P_37+/25-_=0.260, P_37-/25+_=0.315, P_37-/25-_=0.416, P_25+/25-_=1.000). The immunofluorescence staining results in **Figure 8B** showed that no statistical difference in CN existed among the four groups (P_37+/37-_=0.469, P_37+/25+_=0.886, P_37+/25-_=0.818, P_37-/25+_=0.575, P_37-/25-_=0.623, P_25+/25-_=0.936). NSM (P_37+/37-_=0.789, P_37+/25+_=0.001, P_37+/25-_=0.001, P_37-/25+_=0.001, P_37-/25-_=0.001, P_25+/25-_=0.897) and NR (P_37+/37-_=0.820, P_37+/25+_<0.001, P_37+/25-_<0.001, P_37-/25+_=0.001, P_37-/25-_<0.001, P_25+/25-_=0.903) in 37+ and 37- were both significantly higher than those in the other two groups, with no difference between 37+ and 37-. Thus, 25°C treatment after enucleation had adverse effects on the spindle morphology and γ-tubulin localization in mice oocytes reconstructed *via* ST, which had nothing to do with Ca^2+^. The optimum operating condition for electrofusion ST technology were the 37°C group without Ca^2+^.

## Discussion

Mitochondrial disease and senile infertility, especially those with aging oocytes, are both closely related to mitochondrial dysfunction (30–39). Currently, the treatment methods mainly include complementary therapies such as the supplement of Coenzyme Q10, NAD, Growth Hormone and other substances that can enhance mitochondrial function (40–43), mitochondrial transfer from aged adipose-derived stem cells (44) and autologous mitochondrial transfer (45, 46), which can only temporarily relieve symptoms, while ST can fundamentally eliminate the influence of abnormal mitochondria. Thus, ST is considered to be the most valuable therapeutic strategy for clinical transformation.

If ST can be used in the clinic, it will bring a ray of hope for patients with mitochondrial genetic diseases, also for patients with senile infertility, especially those with aging oocytes, and the key is to prove the safety and effectiveness of ST technology. Compared with human oocytes and non-human primate oocytes, mice oocytes are easier to obtain and can be used for large-scale experiments. Therefore, mice oocytes were used as the model to clarify the factors and mechanism for chromosome abnormality in oocytes reconstructed *via* ST. In this study, we demonstrated that electrofusion stimulation was an independent factor of chromosome abnormality in mice oocytes reconstructed *via* ST, and that it was unrelated to enucleation, fusion status, temperature, and Ca^2+^. Electrofusion stimulation could induce premature chromosome separation and abnormal spindle morphology and assembly by decreasing the MPF activity, leading to premature activation, and thus resulting in chromosome abnormality in mice oocytes reconstructed *via* ST. The optimum operating conditions for electrofusion ST was found to be the 37°C group without Ca^2+^.

In order to explore whether premature activation occurred in the ST process and in which procedure (enucleation, electrofusion stimulation), we first detected the MPF activity in the ST process. No significant difference in MPF existed between pre-ST and Ctrl, while MPF activity significantly decreased in the Unfused ST and ST group, with the decline being most significant in the ST group. During the enucleation process, chromosomes were arranged in the center of the spindle and the spindle morphology was normal, showing a typical bipolar, symmetrical, and spindle-shaped structure, with γ-tubulin located at both poles of the spindle. However, after electrofusion stimulation, CN, NSM, NR in the unfused group and the fused group were all significantly reduced, which is consistent with previous studies (4, 47, 48). Based on these observations, we predicted that it was electrofusion stimulation, whether fused or not, rather than enucleation, that caused MPF inactivation and abnormal chromosome activity and spindle organization during meiosis, leading to premature activation.

To further explore the induction of premature activation, we subdivided electrofusion into three groups (SEF, DEF, and TEF). Culture results showed that the fusion rate in DEF and TEF was significantly higher than that in SEF, but in TEF the blastocyst rate decreased significantly, so did the MPF activity, CN, NSM and NR, with no significant difference between Ctrl, SEF and DEF, concluding that single or double electrofusion had little effect on mice oocytes reconstructed *via* ST, while triple electrofusion might have a negative effect, and that a threshold might exist in MPF inactivation. Besides, a precise spindle assembly is the guarantee for the normal separation of chromosomes, especially the spindle pole assembly, spindle morphology, and length of the spindle (49, 50). As the electrofusion stimulation number increased, several abnormalities were generated, especially in TEF. Thus, increasing the electrofusion stimulation number could promote the fusion of ST reconstructed oocytes, but the electrofusion stimulation number should be minimized, with no more than 2 times being appropriate.

Next, we directly stimulated MII oocytes with different electrofusion. The results showed that electrofusion stimulation also resulted in premature activation in MII oocytes, especially in the three shock group (TEF) and the low intensity multiple shock group (4*MII-DEF1/3). So premature activation had nothing to do with fusion state, and electrofusion stimulation was the key factor in triggering premature activation. In addition, the blastocyst rate was significantly reduced in the MII-TEF group, but there was no statistical difference in the chromosome abnormality rate in blastocyst among the 4 groups. We hypothesized that the blastocyst culture process would eliminate embryos with chromosomal abnormalities in the MII-TEF group, so it would significantly decrease the number of normal embryos and reduce the availability of embryos.

To explore whether there was a correlation between the temporary disappearance of the spindle and inhibition of premature activation, ST reconstructed oocytes were treated at 37°C and 25°C for 5 min before electrofusion, respectively, and fused oocytes were used for immunofluorescence staining after recovery for 30 minutes. There was no difference in CN between the two groups, but NSM and NR in the 25°C group were significantly lower, along with the fertilization rate, which indicated that room temperature treatment before electrofusion did not inhibit premature activation, and might affect spindle function in ST reconstructed oocytes.

Furthermore, we investigated the effect of a Ca^2+^ free medium on electrofusion ST reconstructed oocytes in mice. MPF activity in the Ca^2+^ group and the Ca^2+^ free group both decreased, and no statistical difference existed in the CN, NSM, and NR. Thus, Ca^2+^ did not inhibit premature activation. Studies have also found that a rise in intracellular Ca^2+^ is caused by intracellular Ca^2+^ release and that Ca^2+^ shock waves are not affected by external Ca^2+^ (3). Oocyte activation cannot be initiated by a single Ca^2+^ rise and its propagation is mediated by Ca^2+^ induced calcium release (CICR) (3). Interestingly, the fusion rate in the Ca^2+^ free group was significantly higher, and the mechanism behind this requires further study. All the above indicated that a Ca^2+^ free manipulation medium did not inhibit premature activation, and that extracellular Ca^2+^ might not be the key factor causing calcium oscillations in oocyte activation. Electrofusion stimulation might induce premature activation in the reconstructed oocytes by changing the open-close state of calcium channels and the regulatory pathway of CICR (51–54).

To further optimize ST technology, we conducted a cross experiment. The NSM and NR in 37+ and 37- were both significantly higher than those in the other two groups. Meanwhile the fusion rate was the highest in 37-, the fertilization rate in 25- was the lowest, and there was no difference in the fertilization rate between 37+, 37-, and 25+. In addition, there was no statistical difference in the developmental potential among the groups. Therefore, the optimum operating condition for electrofusion ST technology were determined to be the 37°C group without Ca^2+^.

In conclusion, the present study revealed that electrofusion stimulation was an independent factor for chromosome abnormality in mice oocytes reconstructed *via* ST, and that it was unrelated to enucleation, fusion status, temperature, and Ca^2+^. Electrofusion stimulation could induce premature chromosome separation and abnormal spindle morphology and assembly by decreasing MPF activity, leading to premature activation, and thus resulting in chromosome abnormality in mice oocytes reconstructed *via* ST. The optimum operating condition for electrofusion ST were determined to be the 37°C group without Ca^2+^.

## Data Availability Statement

The original contributions presented in the study are included in the article/**Supplementary Material**. Further inquiries can be directed to the corresponding authors.

## Ethics Statement

This study was reviewed and approved by the Institutional Animal Care and Use Committee of the Sixth Medical Center of China PLA General Hospital (HZKY-PJ-2019-3).

## Author Contributions

WW, WS, and LZ conceived and designed the experiments. WW and YL performed the experiments. WW, SS, WC, WZW, and YC analyzed the data. WW drafted the article. WW, YG, SH, MS, and QW prepared the digital images. All authors contributed to the article and approved the submitted version.

## Funding

This study was funded by the National Key Research and Development Program of China (No. 2018YFC1003003).

## Conflict of Interest

The authors declare that the research was conducted in the absence of any commercial or financial relationships that could be construed as a potential conflict of interest.

## Publisher’s Note

All claims expressed in this article are solely those of the authors and do not necessarily represent those of their affiliated organizations, or those of the publisher, the editors and the reviewers. Any product that may be evaluated in this article, or claim that may be made by its manufacturer, is not guaranteed or endorsed by the publisher.

## Abbreviations

ST, Spindle transfer; CB, Cytochalasin B; MPF, Maturation-Promoting Factor; RIPA, radio immunoprecipitation assay; Pb1, first polar body; sp, spindle;Ctrl, the control group; pre-ST, enucleation oocytes; Unfused ST, unfused ST reconstructed oocytes; CN, chromosomal nondisjunction rate; NSM, normal spindle morphology rate; NR, normal g-tubulin rate; SEF, the single electrofusion group; DEF, the double electrofusion group; TEF, the triple electrofusion group; MII-SEF, the single electrofusion group in MII oocytes; MII-DEF, the double electrofusion group in MII oocytes; MII-TEF, the triple electrofusion group in MII oocytes; SCNT, somatic cell nuclear transfer; MTOC, the microtubule organizing center; CICR, Ca2^+^ induced calcium release; SSS, Synthesis Serum Subtituent; PVA, polyvinyl alcohol.
