# Supplementary material for: Electrofusion Stimulation Is an Independent Factor of Chromosome Abnormality in Mice Oocytes Reconstructed via Spindle Transfer
Source: Front Endocrinol (Lausanne). 2021 Jul 28;12:705837. doi: 10.3389/fendo.2021.705837 (PMC8370092; doi:10.3389/fendo.2021.705837)

Supplementary Material

**Supplemental figure S1** The spindle transfer process in mice oocytes: (A) Adjusted oocytes, showing the spindle; (B) Enucleation. The spindle was gently aspirated into the micromanipulation needle; (C) The extracted spindle; (D-E) Nucleus injection. The spindle was transferred into the perivitelline space of an enucleated donor cytoplast; (F) The reconstructed oocyte: After electrofusion, the spindle had fused with the donor cytoplast. The arrow shows the spindle. A, B and F were taken by polariscope; the rest were taken under brightfield. Scale bar, 20μm.


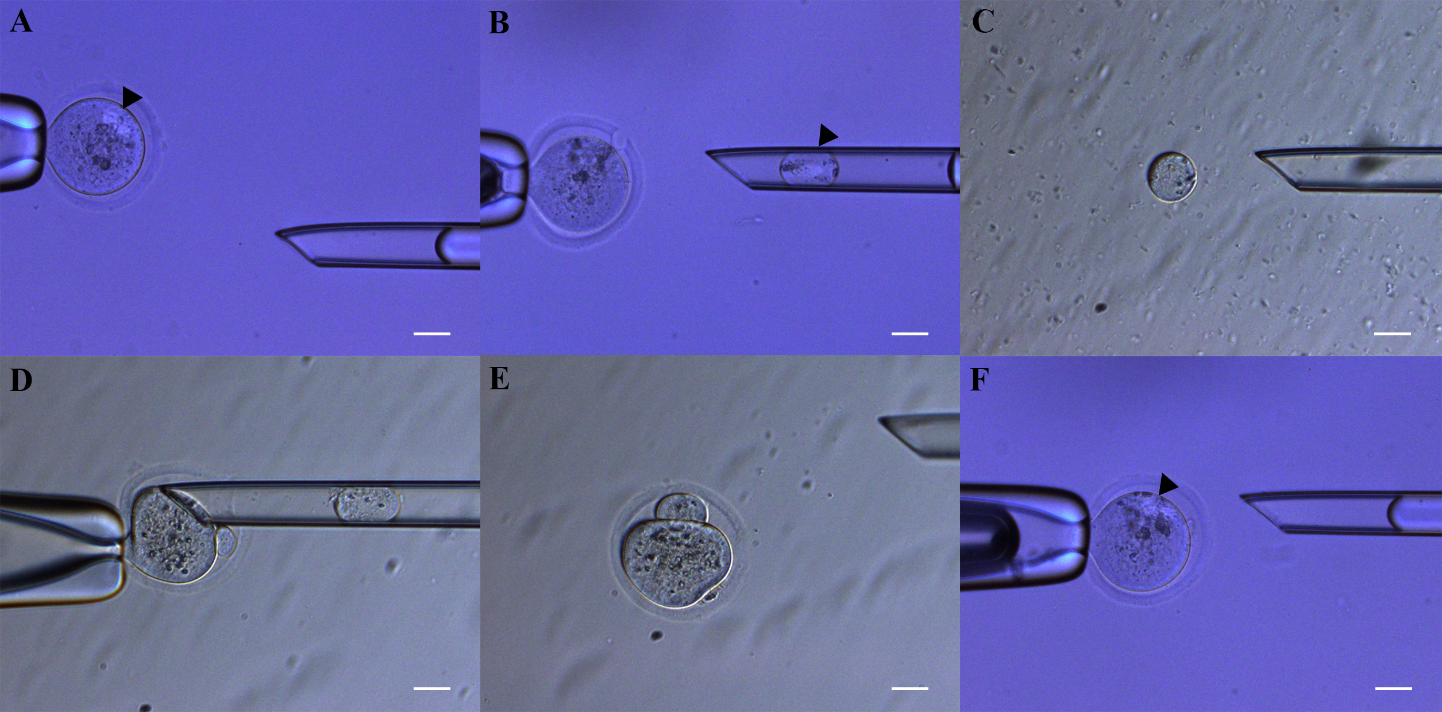


**Supplemental Table S1**

The number of mice, oocytes and replications used in each group

| Figure | Group | Mice(n) | Oocytes(n) | Replications(n) |
| --- | --- | --- | --- | --- |
| Figure 1A | Ctrl | 36 | 103 | 3 |
|  | Pre-ST |  | 111 | 3 |
|  | Unfused ST |  | 136 | 3 |
|  | ST |  | 145 | 3 |
| Figure 1BC | Ctrl | 20 | 46 | 4 |
|  | Pre-ST |  | 38 | 4 |
| Figure 1DE | Pre-ST |  | 38 | 4 |
|  | Unfused ST |  | 38 | 4 |
|  | ST |  | 32 | 4 |
| Figure 2A | Ctrl | 46 | 79 | 8 |
|  | SEF |  | 205 | 8 |
|  | DEF |  | 249 | 8 |
|  | TEF |  | 219 | 8 |
| Figure 2B | Ctrl | 30 | 92 | 3 |
|  | SEF |  | 89 | 3 |
|  | DEF |  | 105 | 3 |
|  | TEF |  | 89 | 3 |
| Figure 2CD | SEF | 20 | 33 | 5 |
|  | DEF |  | 53 | 5 |
|  | TEF |  | 80 | 5 |
| Figure 4A | Ctrl | 34 | 78 | 3 |
|  | MII-SEF |  | 84 | 3 |
|  | MII-DEF |  | 85 | 3 |
|  | MII-TEF |  | 83 | 3 |
| Figure 4BE | MII-SEF | 12 | 38 | 3 |
|  | MII-DEF |  | 36 | 3 |
|  | MII-TEF |  | 35 | 3 |
| Figure 4C | Ctrl | 34 | 78 | 3 |
|  | MII-DEF |  | 85 | 3 |
|  | 2*MII-DEF1/2 |  | 84 | 3 |
|  | 3*MII-DEF1/3 |  | 76 | 3 |
|  | 4*MII-DEF1/3 |  | 80 | 3 |
| Figure 4DE | 2*MII-DEF1/2 | 15 | 37 | 3 |
|  | 3*MII-DEF1/3 |  | 37 | 3 |
|  | 4*MII-DEF1/3 |  | 35 | 3 |
| Figure 5A | Ctrl | 47 | 79 | 3 |
|  | MII-SEF |  | 127 | 3 |
|  | MII-DEF |  | 134 | 3 |
|  | MII-TEF |  | 100 | 3 |
| Figure 5BC | Ctrl | 48 | 19(blastocyst) | 3 |
|  | MII-SEF |  | 15(blastocyst) | 3 |
|  | MII-DEF |  | 14(blastocyst) | 3 |
|  | MII-TEF |  | 19(blastocyst) | 3 |
| Figure 6A | 37°C | 50 | 342 | 10 |
|  | 25°C |  | 126 | 10 |
| Figure 6BC | 37°C | 13 | 44 | 3 |
|  | 25°C |  | 36 | 3 |
| Figure 7A | +Ca^2+^ | 51 | 330 | 10 |
|  | -Ca^2+^ |  | 138 | 10 |
| Figure 7B | Ctrl | 30 | 92 | 3 |
|  | +Ca2+ |  | 95 | 3 |
|  | -Ca2+ |  | 97 | 3 |
| Figure 7C | +Ca^2+^ | 12 | 44 | 3 |
|  | -Ca^2+^ |  | 30 | 3 |
| Figure 8A | 37+ | 20 | 147 | 4 |
|  | 37- |  | 79 | 4 |
|  | 25+ |  | 67 | 4 |
|  | 25- |  | 59 | 4 |
| Figure 8B | 37+ | 14 | 44 | 3 |
|  | 37- |  | 36 | 3 |
|  | 25+ |  | 30 | 3 |
|  | 25- |  | 38 | 3 |

**Supplemental table S2**

Karyotype analysis results for blastocysts among the 4 groups

(Ctrl,MII-SEF,MII-DEF, MII-TEF)

| **NO.** | **Sample number** | **Serial number** | **Reads** | **Compare reads** | **CNVs** | **Gender** |
| --- | --- | --- | --- | --- | --- | --- |
| 1 | C1 | P210224003P01 | 3592517 | 2532902 | Null | female |
| 2 | C2 | P210224003P02 | 3105231 | 2221597 | Null | female |
| 3 | C3 | P210224003P03 | 3276836 | 2353985 | Null | female |
| 4 | C4 | P210224003P04 | 3703658 | 2585896 | Null | male |
| 5 | C5 | P210224003P05 | 3114136 | 2237429 | Null | female |
| 6 | C6 | P210224003P06 | 3279597 | 2271577 | Null | male |
| 8 | C12 | P210224003P08 | 3603278 | 2559386 | Null | male |
| 9 | C14 | P210224003P09 | 3988224 | 2863409 | Null | female |
| 10 | C15 | P210224003P10 | 4682190 | 3346118 | Null | female |
| 11 | C16 | P210224003P11 | 5367223 | 3739941 | Null | male |
| 12 | C17 | P210224003P12 | 4221783 | 3010764 | Null | male |
| 13 | C18 | P210224003P13 | 4271012 | 3054642 | +7; | female |
| 14 | C19 | P210224003P14 | 3986696 | 2901950 | Null | female |
| 15 | C20 | P210224003P15 | 4702985 | 3379815 | Null | female |
| 46 | C-9 | P210224003P46 | 5132668 | 4191745 | Null | male |
| 47 | C-10 | P210224003P48 | 3880079 | 3257370 | +3;-7;+13;-X; | female |
| 49 | C-21 | P210224003P50 | 4071727 | 3463346 | Null | female |
| 50 | C-22 | P210224003P51 | 4673726 | 3889001 | Null | female |
| 52 | C-24 | P210224003P53 | 3600541 | 3054670 | Null | female |
| 17 | 1-8 | P210224003P17 | 2694563 | 1961416 | Null | female |
| 18 | 1-9 | P210224003P18 | 3709541 | 2724204 | Null | female |
| 19 | 1-10 | P210224003P19 | 3550508 | 2591575 | Null | female |
| 20 | 1-11 | P210224003P20 | 3900320 | 2839283 | Null | female |
| 21 | 1-12 | P210224003P21 | 1983068 | 1641918 | Null | female |
| 23 | 1-14 | P210224003P23 | 2131572 | 1788875 | Null | female |
| 24 | 1-15 | P210224003P24 | 2672513 | 2256911 | Null | female |
| 25 | 1-16 | P210224003P25 | 2968238 | 2444935 | Null | female |
| 26 | 1-17 | P210224003P26 | 2665990 | 2223248 | Null | female |
| 27 | 1-18 | P210224003P27 | 2582502 | 2161052 | Null | female |
| 29 | 1-20 | P210224003P29 | 3455856 | 2883198 | Null | female |
| 30 | 1-21 | P210224003P30 | 3192204 | 2594569 | Null | female |
| 53 | 1-3 | P210224003P54 | 3911448 | 3356161 | '-14; | female |
| 54 | 1-4 | P210224003P55 | 4739523 | 3976856 | '-(mosaic)(7); | female |
| 58 | 1-22 | P210224003P59 | 4202954 | 3544564 | Null | female |
| 63 | 2-1 | P210224003P16 | 4320678 | 3082032 | '+8;+9;+(mosaic)(15); | female |
| 64 | 2-2 | P210224003P49 | 3606297 | 2927144 | -11;+2;dup(12)(p13.32-q12).seq[GRCh37/hg19](3500001-46100000)X3[36.5Mb]; | female |
| 65 | 2-3 | P210406001-07 | 1351530 | 975675 | Null | female |
| 66 | 2-4 | P210406001-11 | 2477583 | 2059824 | Null | female |
| 67 | 2-5 | P210406001-12 | 2449112 | 2072158 | Null | female |
| 68 | 2-6 | P210406001-15 | 4082776 | 3375886 | Null | male |
| 69 | 2-7 | P210406001-18 | 3688555 | 3085673 | Null | female |
| 70 | 2-8 | P210406001-02 | 3105152 | 2221522 | Null | female |
| 71 | 2-9 | P210406001-05 | 3114169 | 2237407 | Null | female |
| 72 | 2-10 | P210406001-06 | 3192213 | 2594661 | Null | male |
| 73 | 2-11 | P210406001-13 | 3606108 | 2927212 | Null | female |
| 74 | 2-12 | P210406001-16 | 3573560 | 2924283 | Null | female |
| 75 | 2-13 | P210406001-17 | 4586391 | 3771899 | Null | female |
| 76 | 2-14 | P210406001-20 | 5183677 | 3024858 | Null | female |
| 31 | 3-1 | P210224003P31 | 3589106 | 2975994 | Null | female |
| 32 | 3-2 | P210224003P32 | 3096792 | 2570647 | Null | female |
| 33 | 3-3 | P210224003P33 | 2308680 | 1943932 | Null | female |
| 34 | 3-5 | P210224003P34 | 2697396 | 2253131 | Null | female |
| 35 | 3-8 | P210224003P35 | 3573442 | 2924220 | Null | female |
| 36 | 3-9 | P210224003P36 | 4586431 | 3771725 | Null | female |
| 37 | 3-10 | P210224003P37 | 3108081 | 2632260 | Null | female |
| 38 | 3-11 | P210224003P38 | 3195938 | 2654439 | Null | female |
| 39 | 3-12 | P210224003P39 | 2531340 | 2121282 | Null | female |
| 40 | 3-14 | P210224003P40 | 3125807 | 2600206 | Null | female |
| 41 | 3-15 | P210224003P41 | 3463198 | 2837594 | Null | female |
| 42 | 3-16 | P210224003P42 | 3711972 | 3091868 | Null | female |
| 43 | 3-17 | P210224003P43 | 3073881 | 2595856 | Null | female |
| 44 | 3-18 | P210224003P44 | 3213997 | 2701177 | Null | female |
| 45 | 3-19 | P210224003P45 | 4121020 | 3434204 | dup(mosaic)(2)(q13-q31.3).seq[GRCh37/hg19](111580001-181420000)X3[64.5Mb]; | female |
| 59 | 3-4 | P210224003P60 | 4063505 | 3437236 | Null | female |
| 60 | 3-6 | P210224003P61 | 5314354 | 4428413 | -3;-11; | female |
| 61 | 3-7 | P210224003P62 | 5183860 | 3024702 | Null | female |
| 62 | 3-20 | P210224003P63 | 4459057 | 3807043 | Null | female |

**Supplemental figure S2**

CNV results for blastocysts among the three groups (Ctrl,MII-SEF, MII-DEF，MII-TEF)

Ctrl


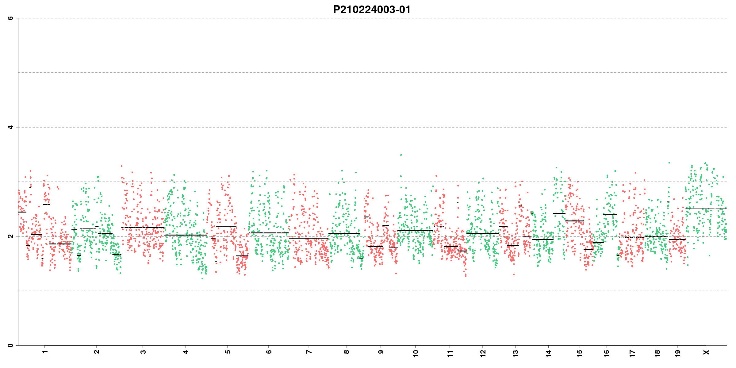


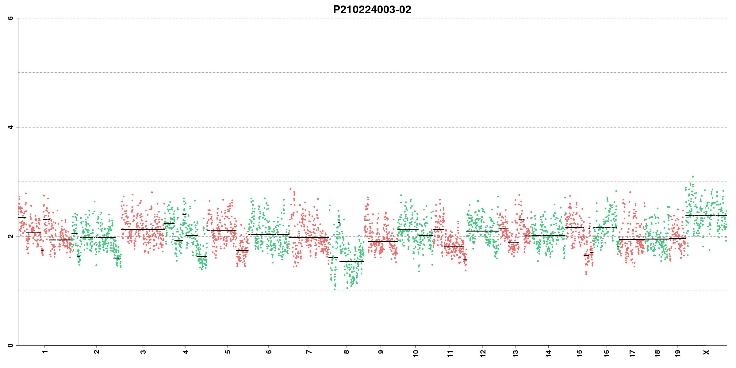


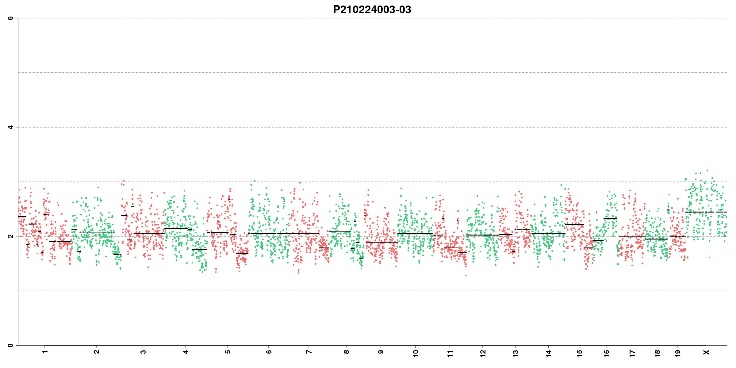


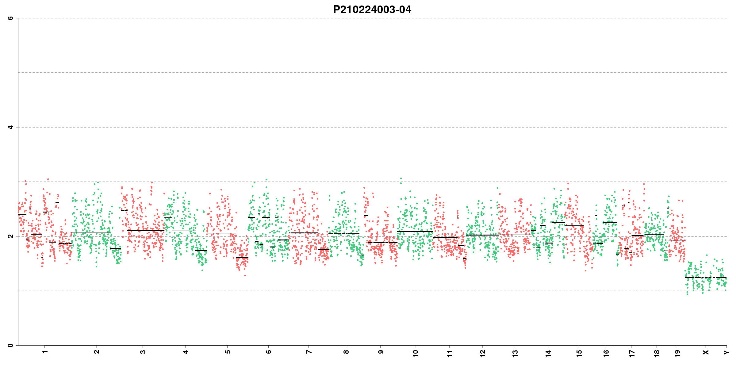


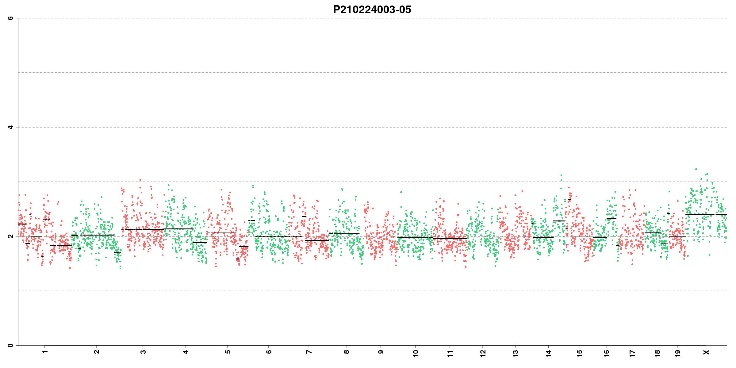


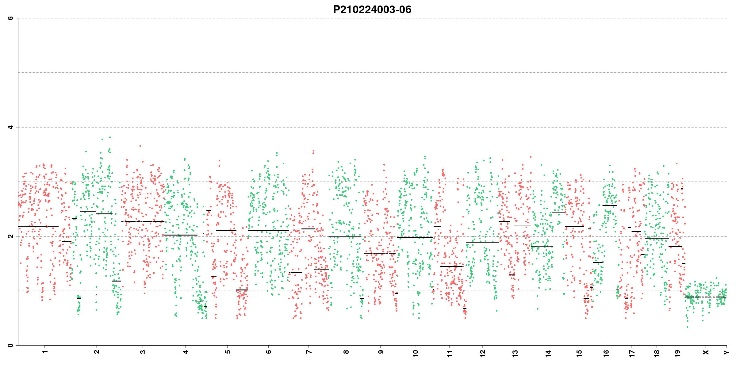


**
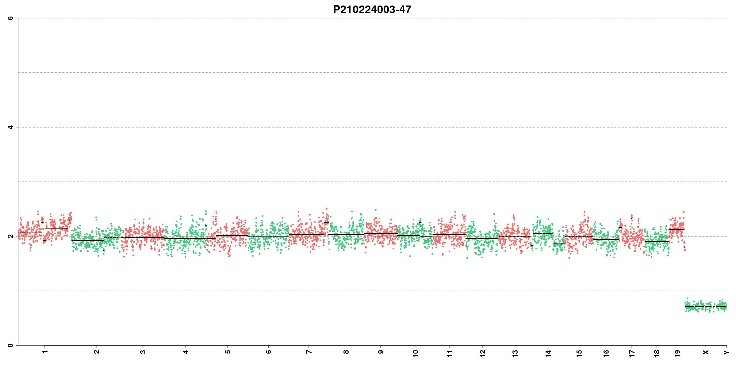
**


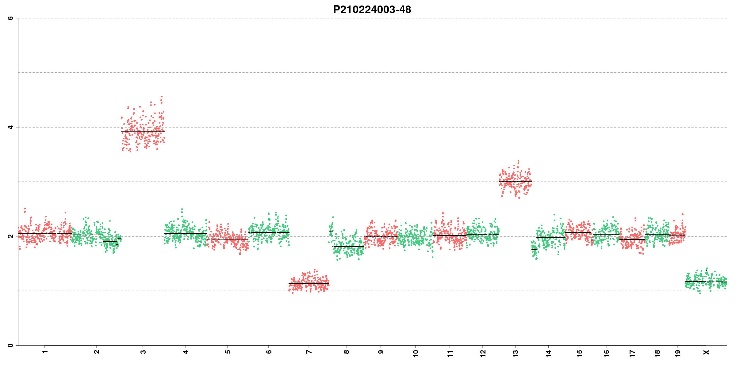


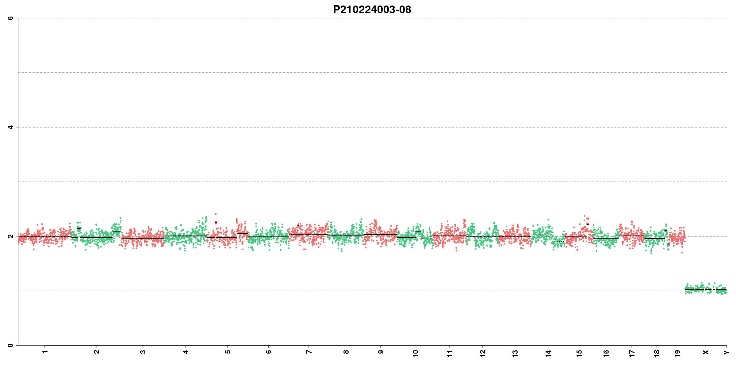


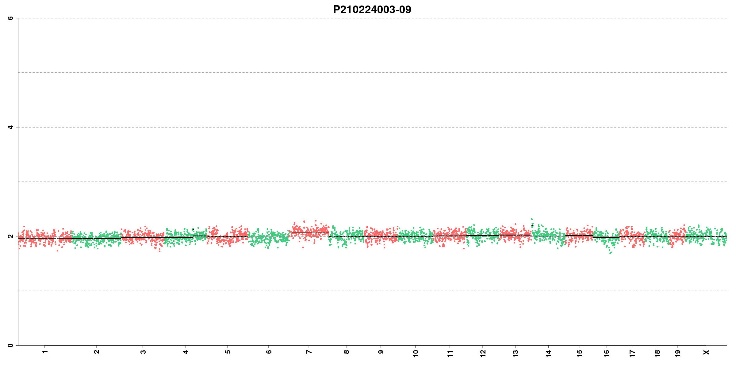


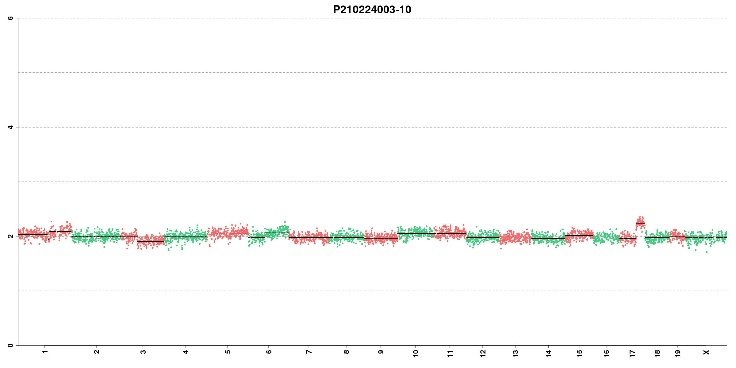


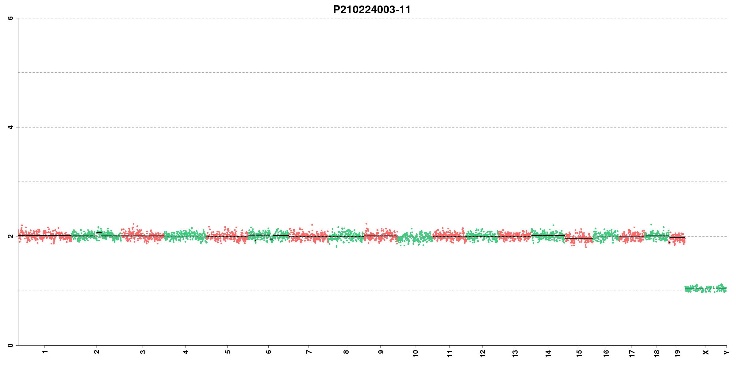


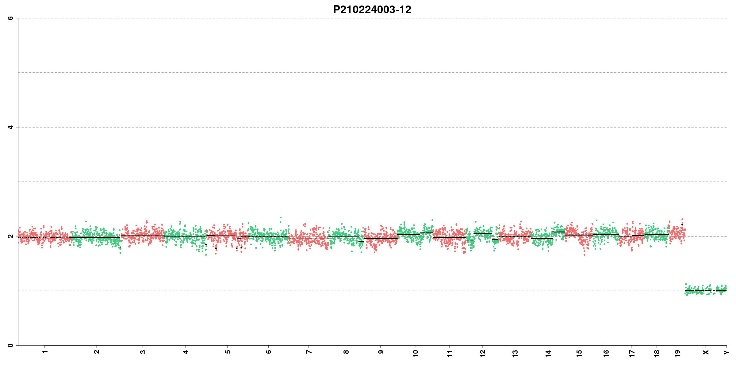


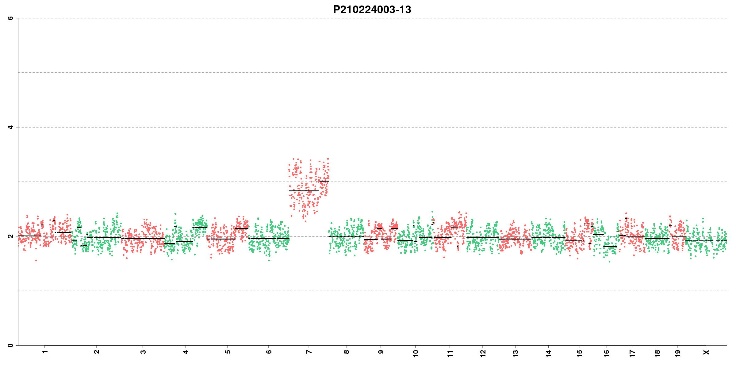


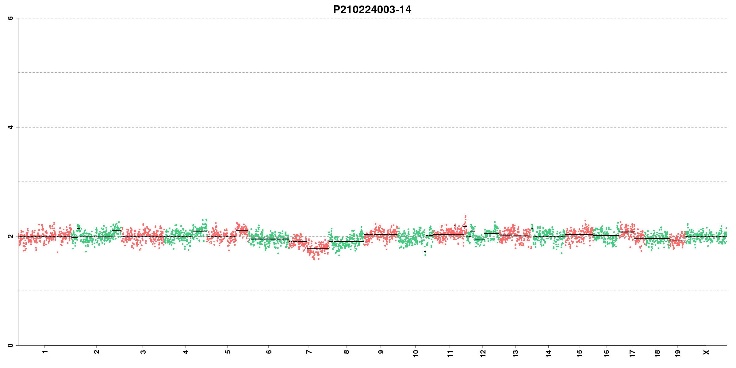


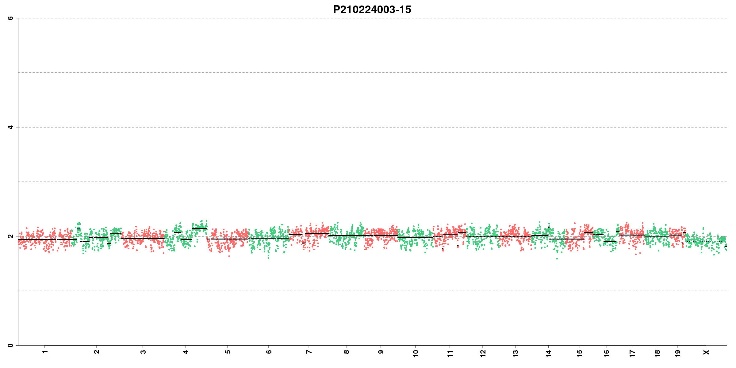


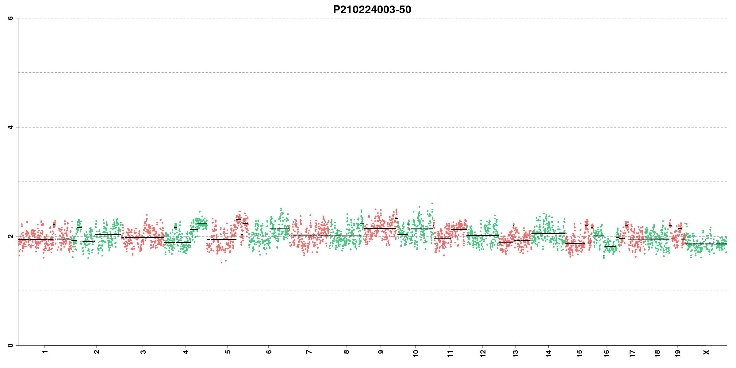


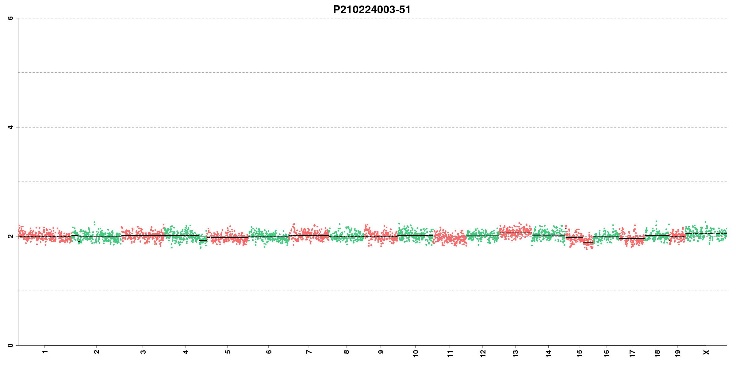


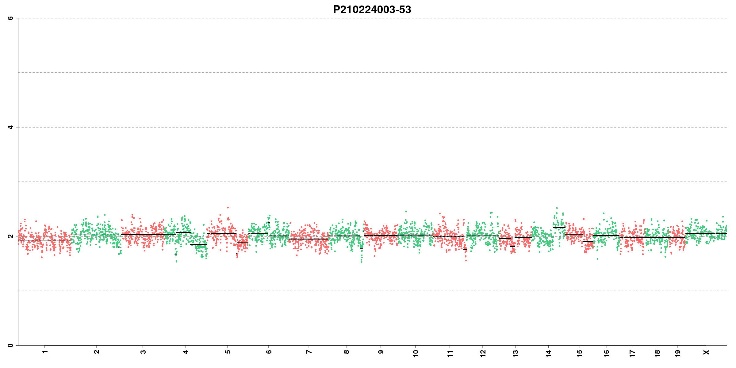


MII-SEF


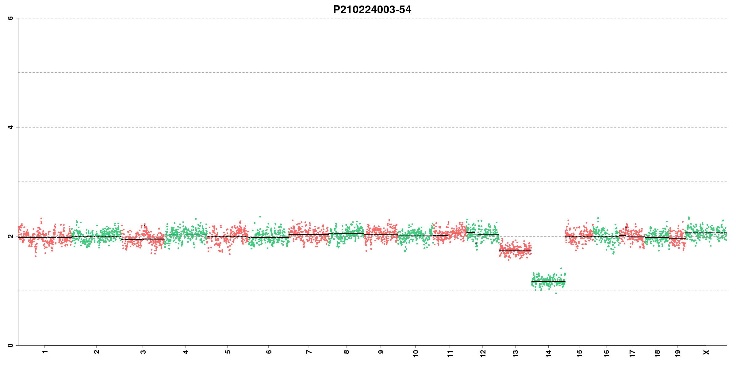


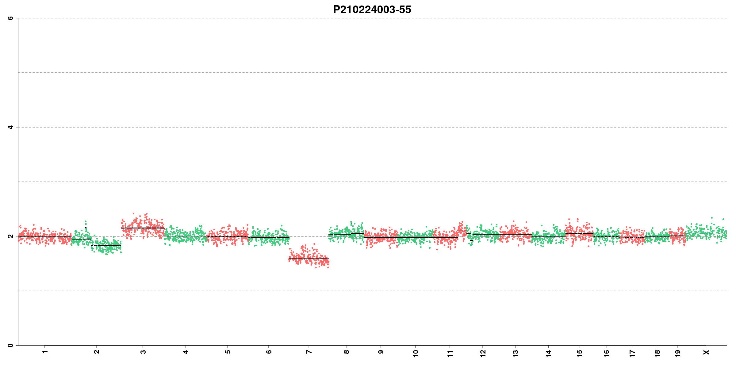


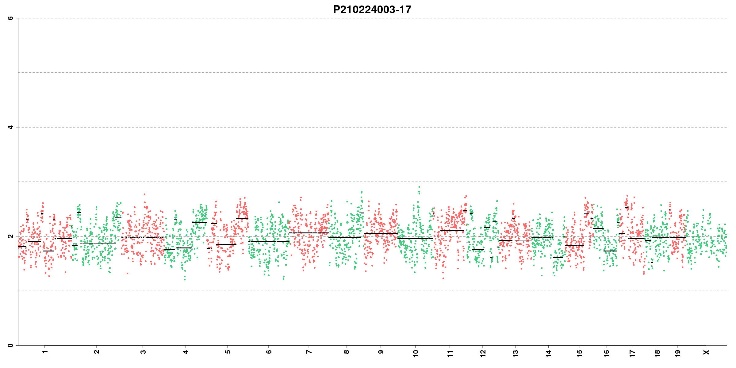


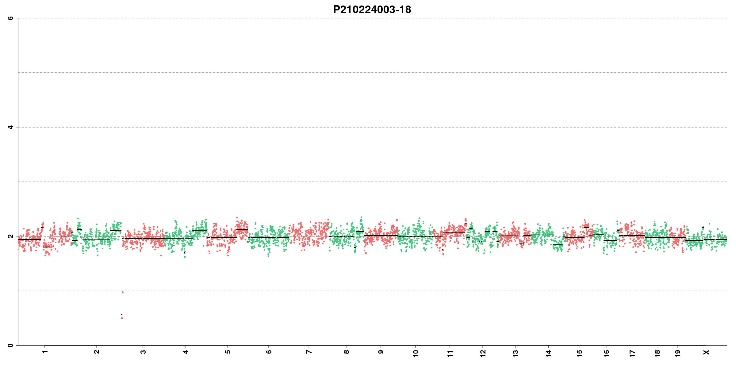


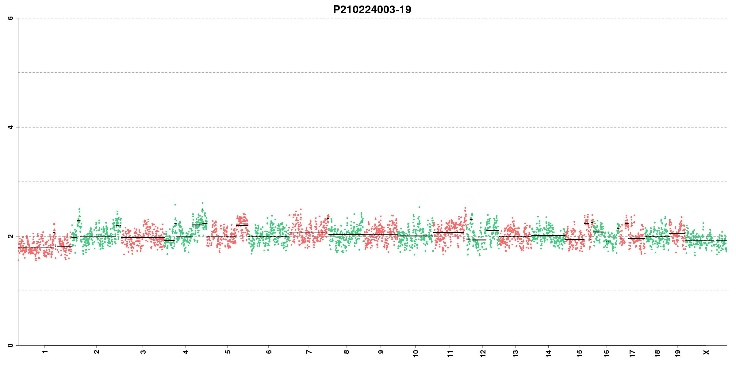


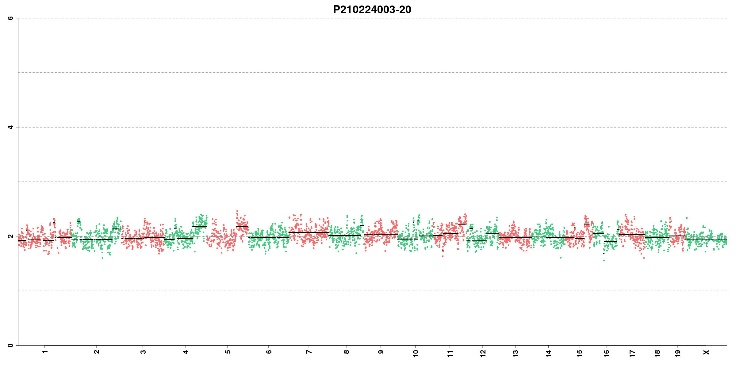


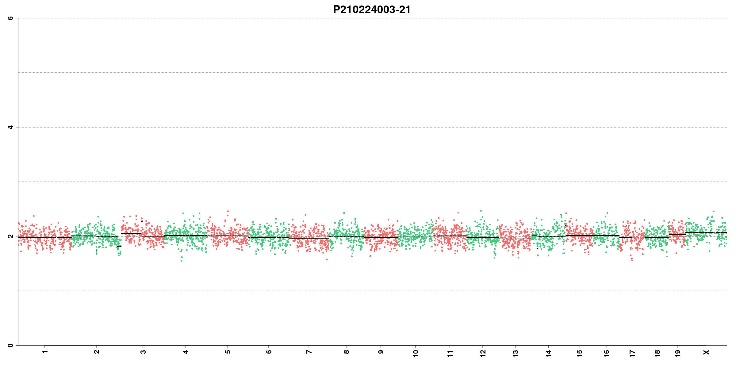


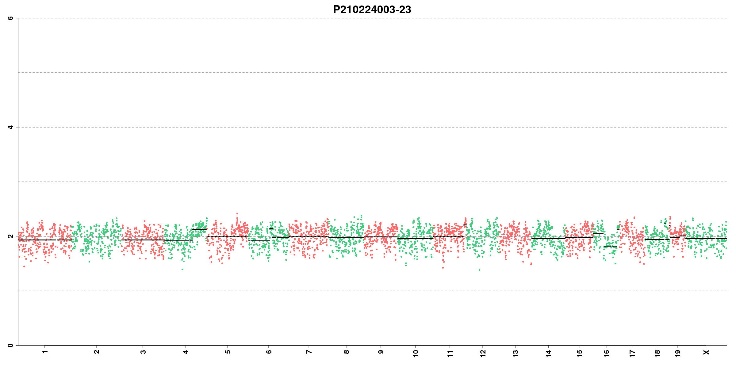


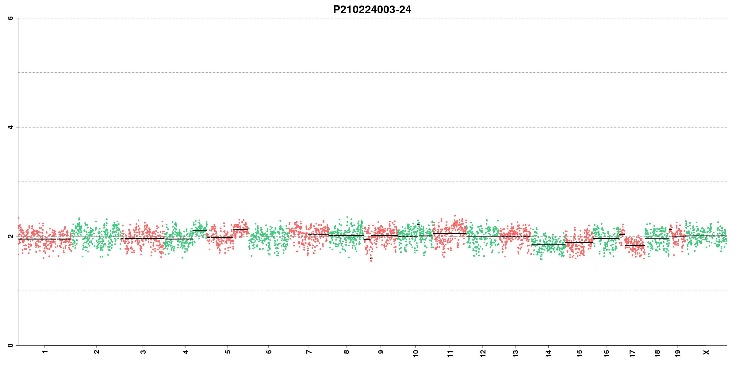


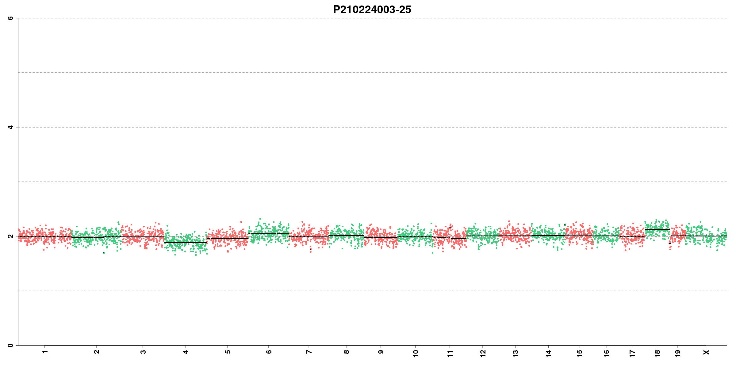


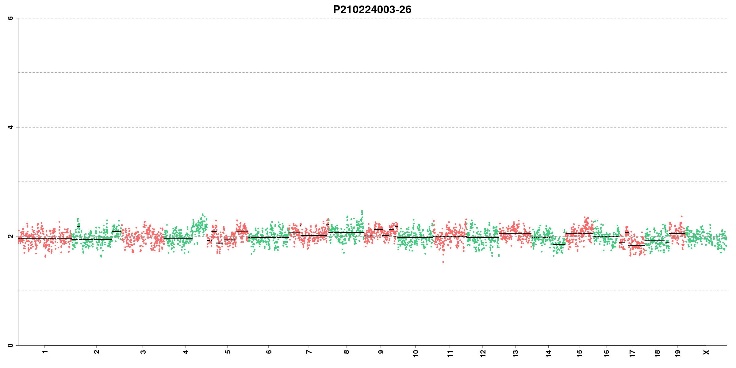


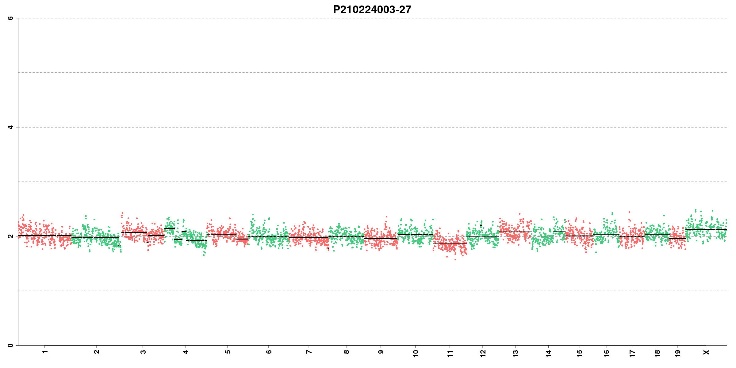


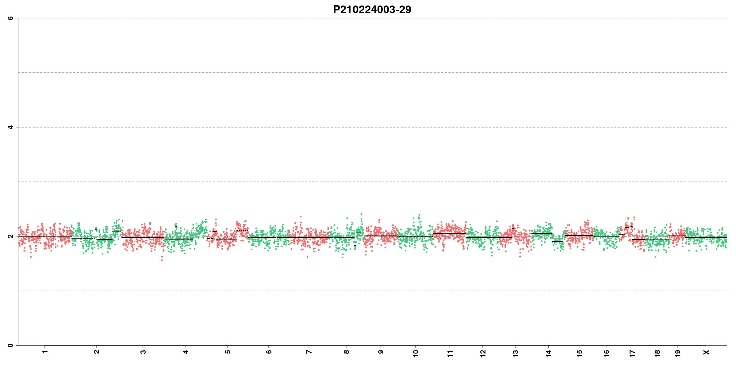


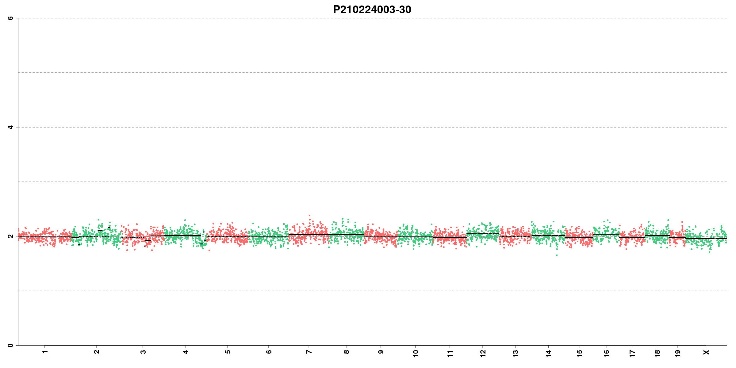


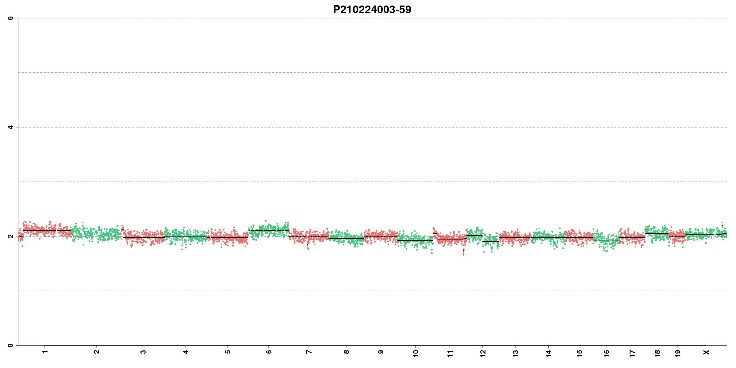


MII-DEF


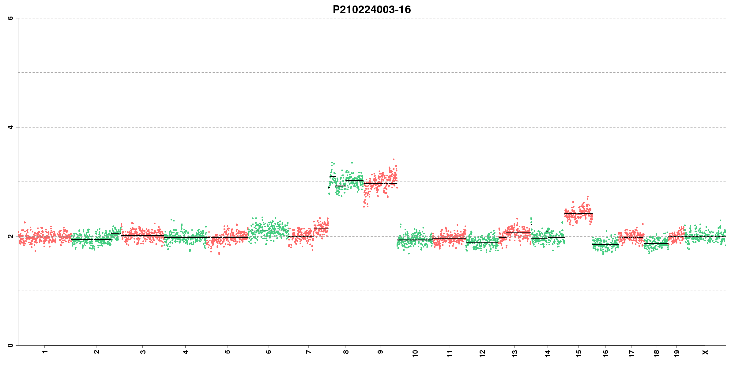


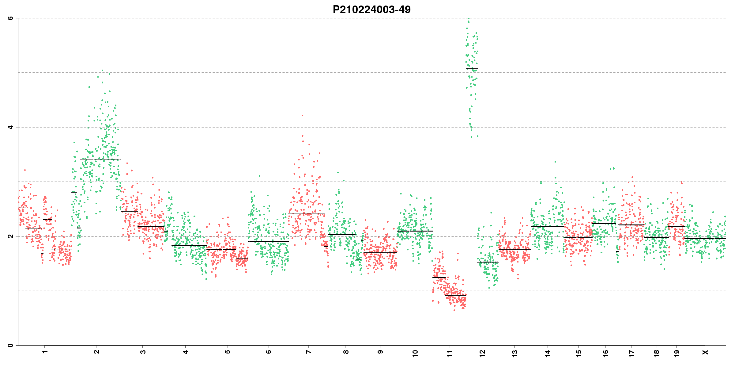


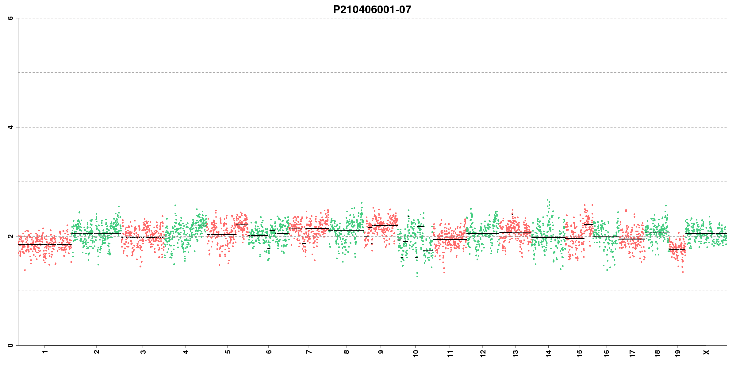


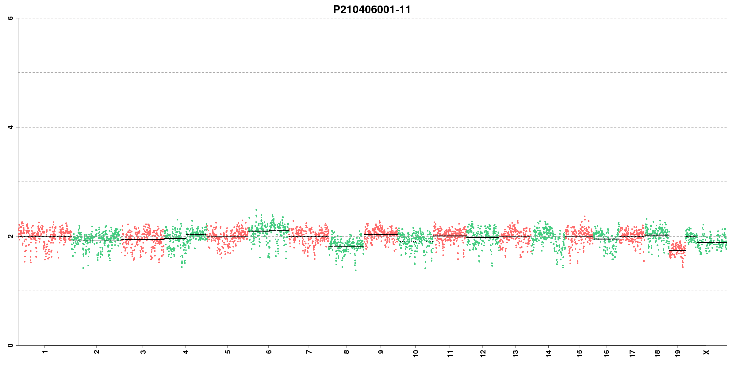


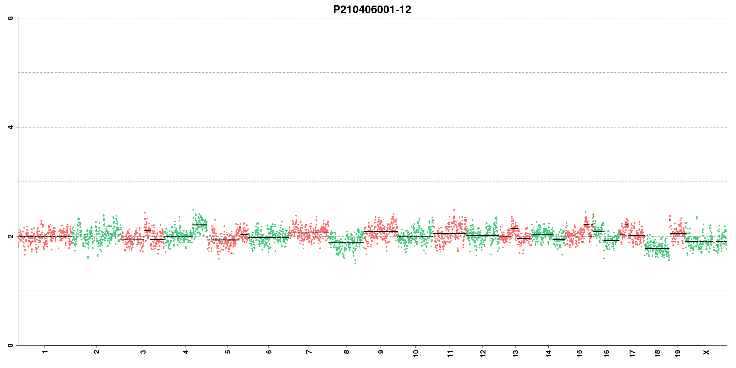


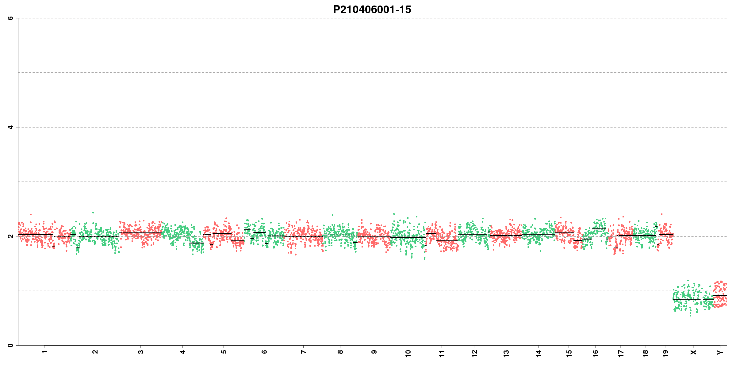


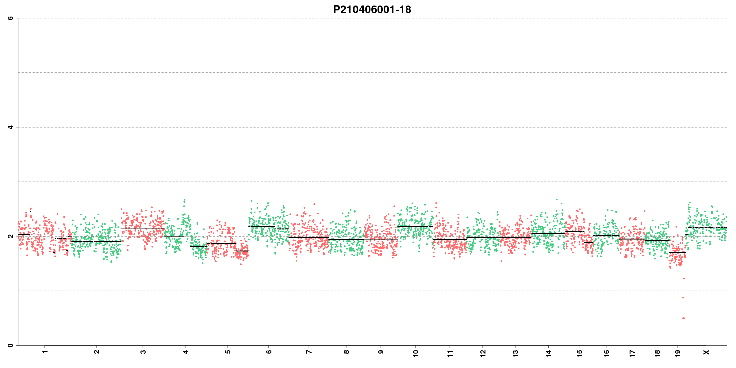


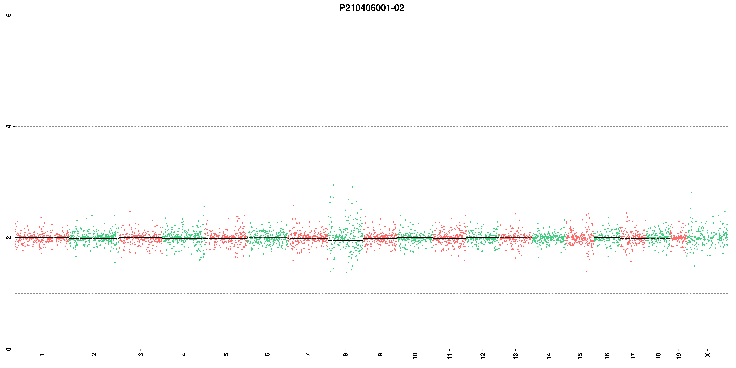


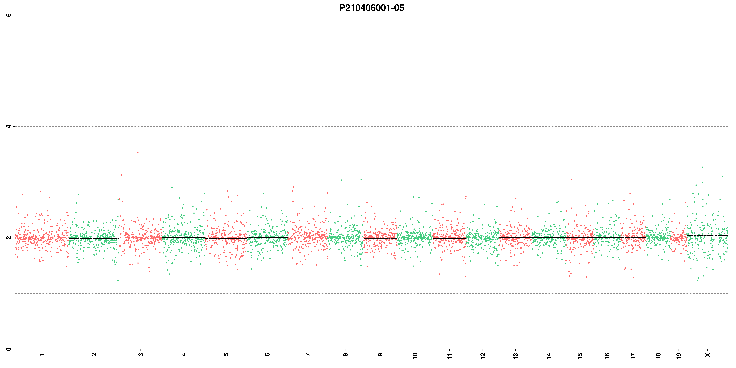


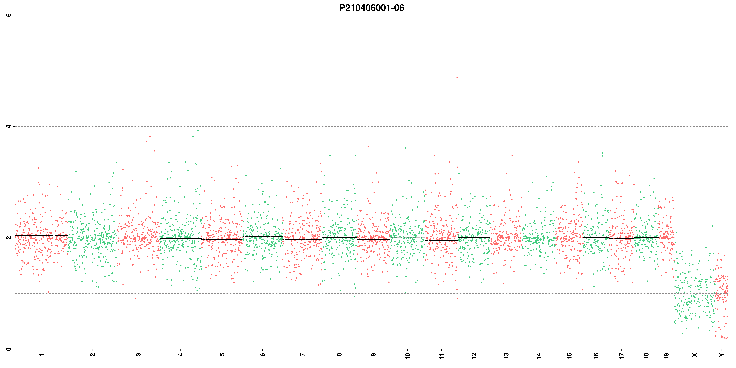


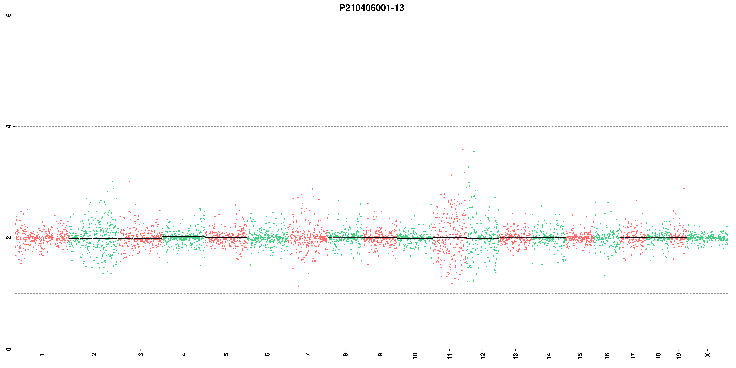


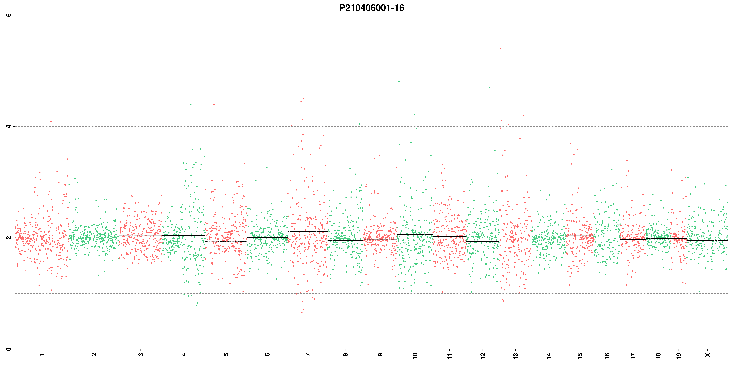


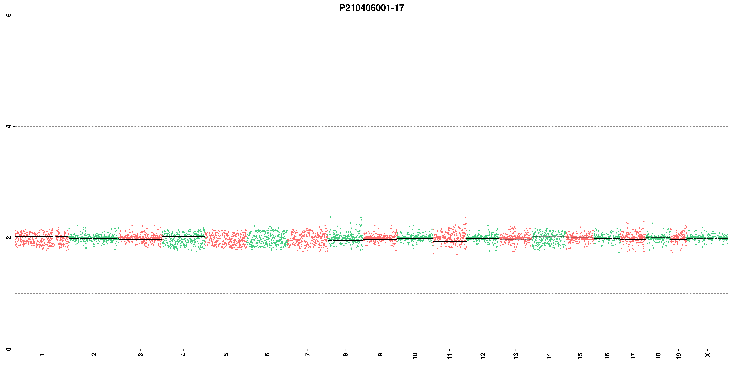


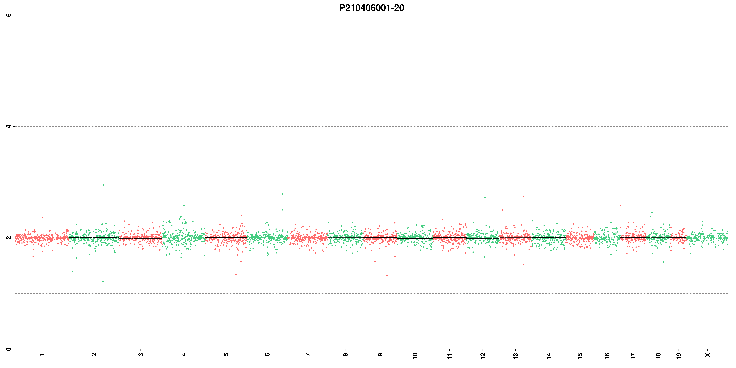


MII-TEF


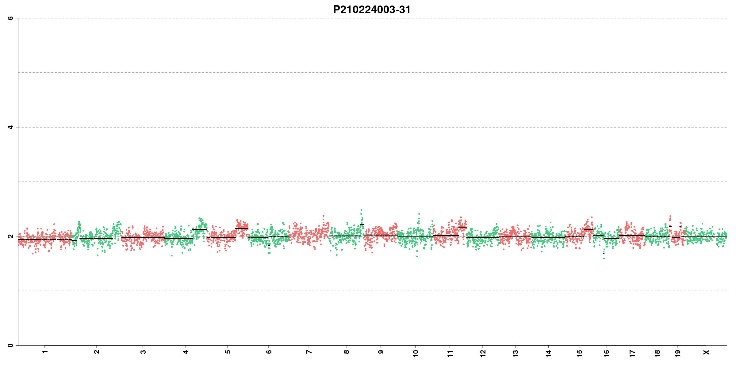


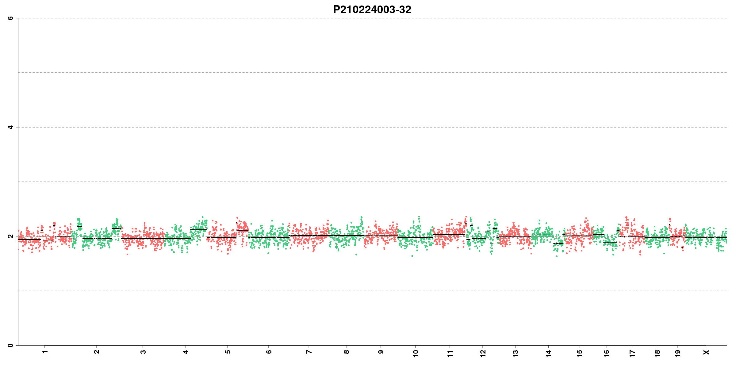


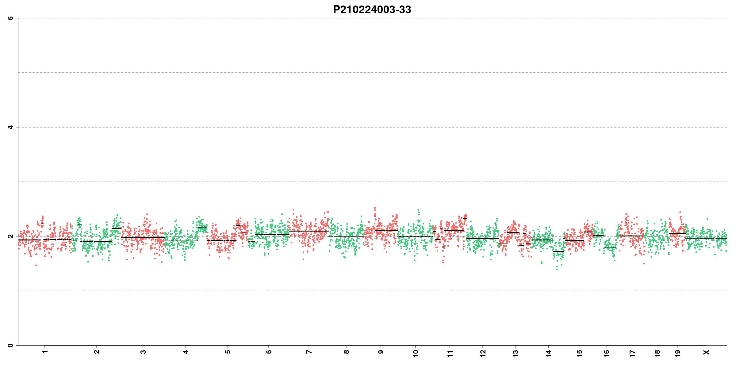


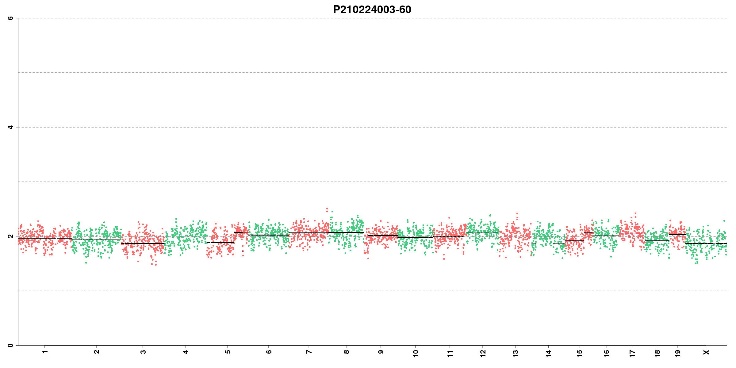


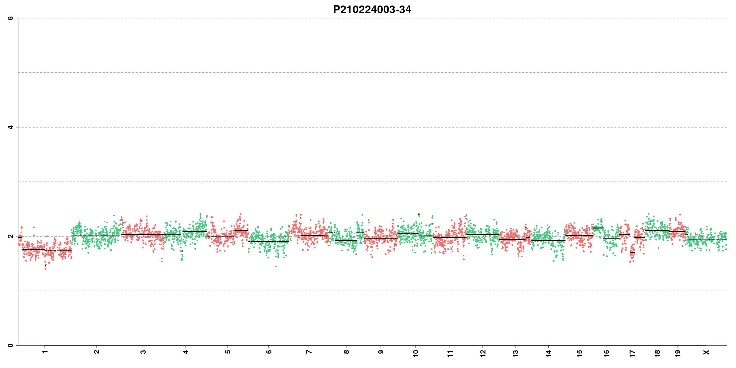


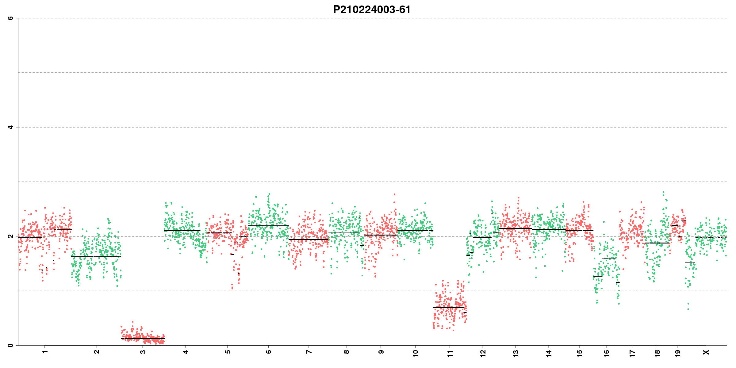


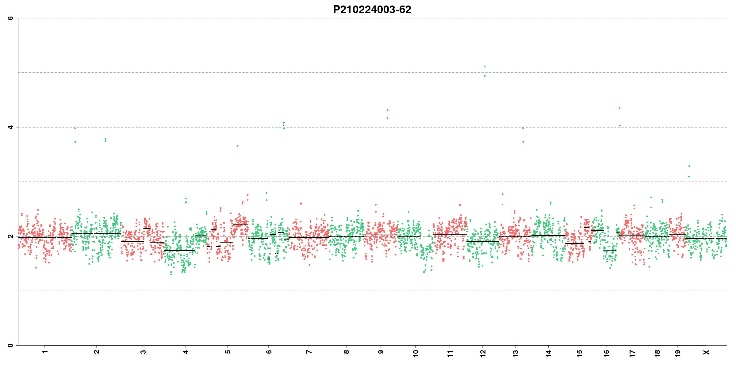


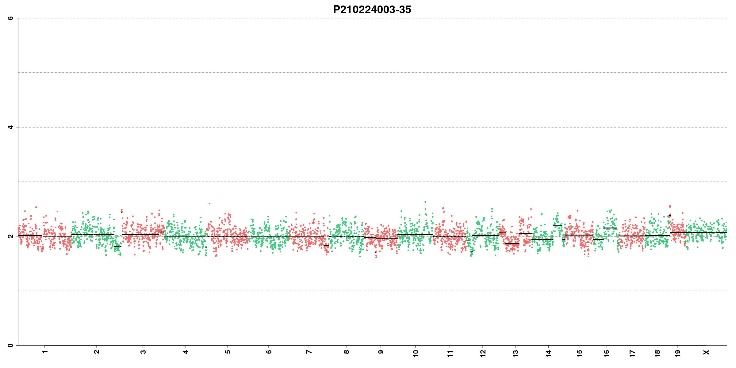


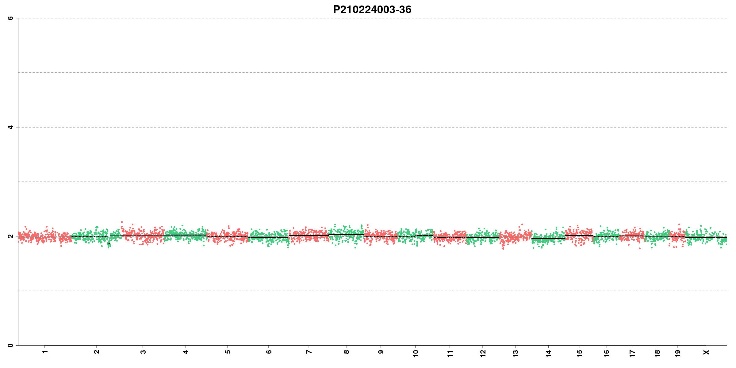


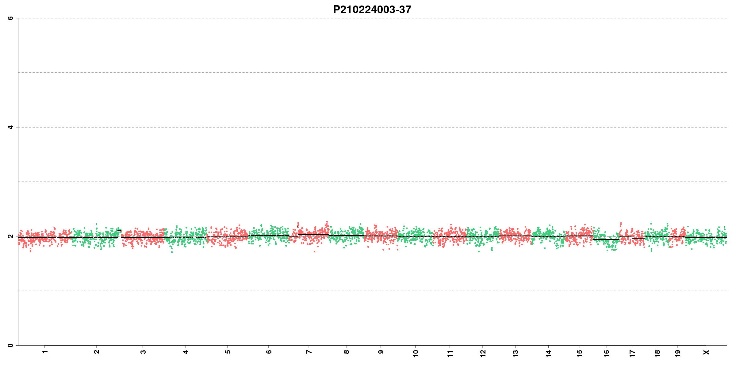


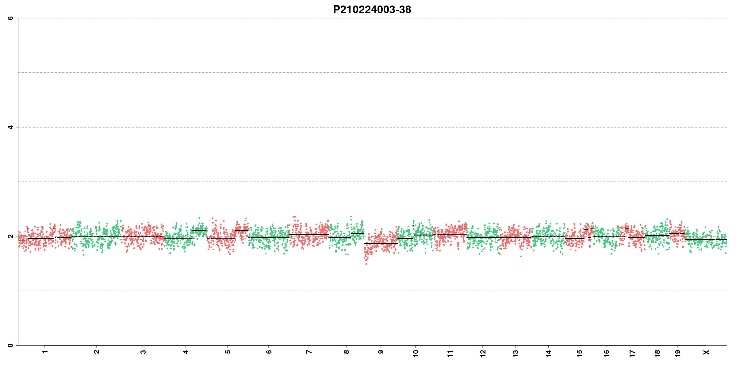


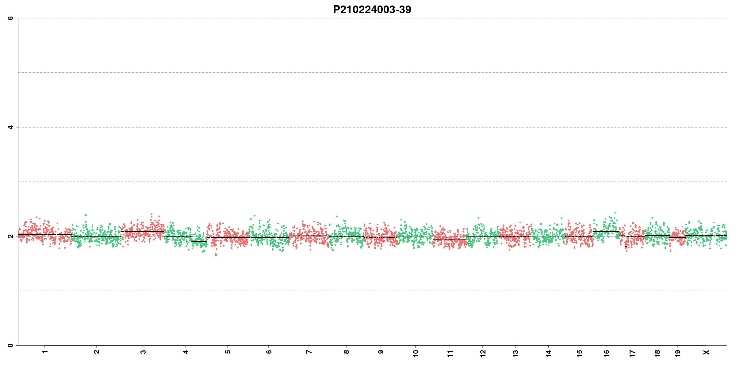


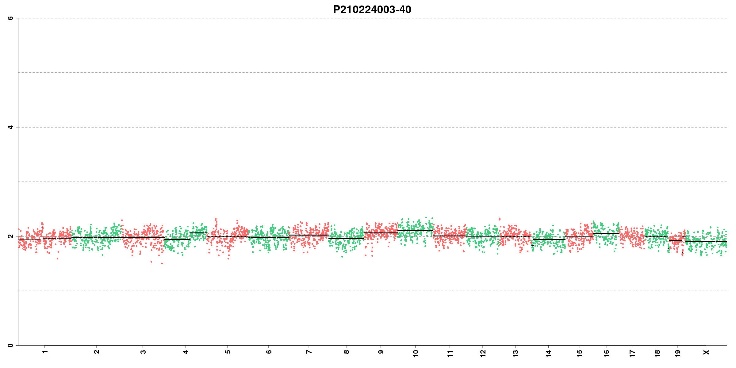


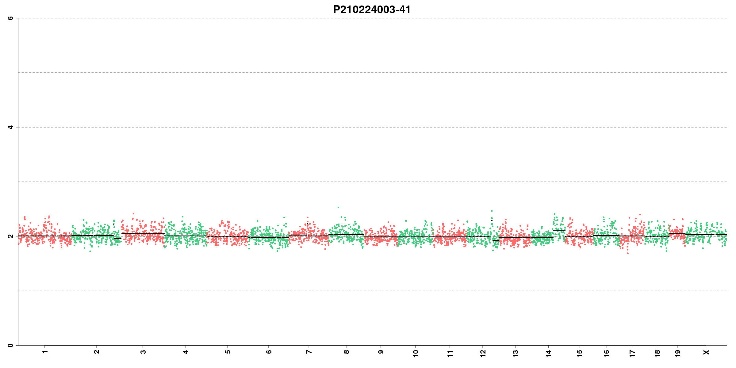


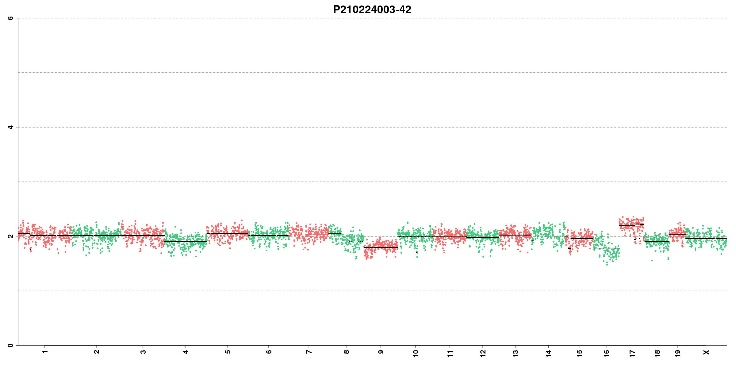


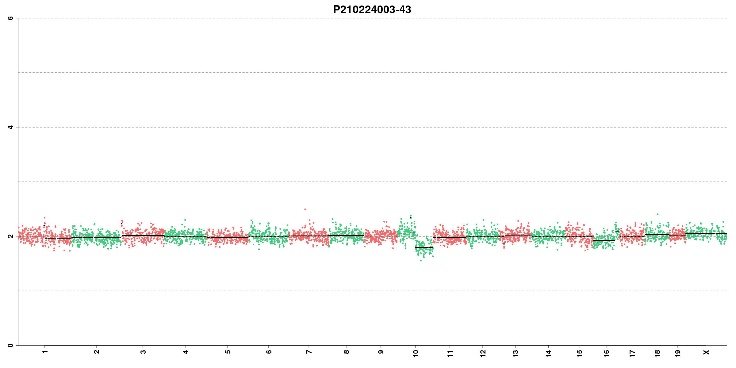


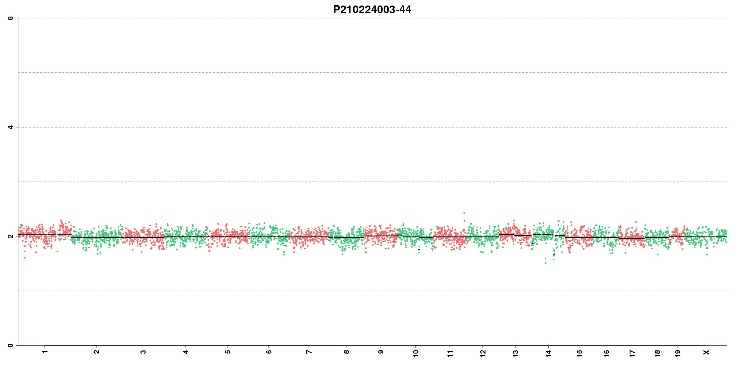


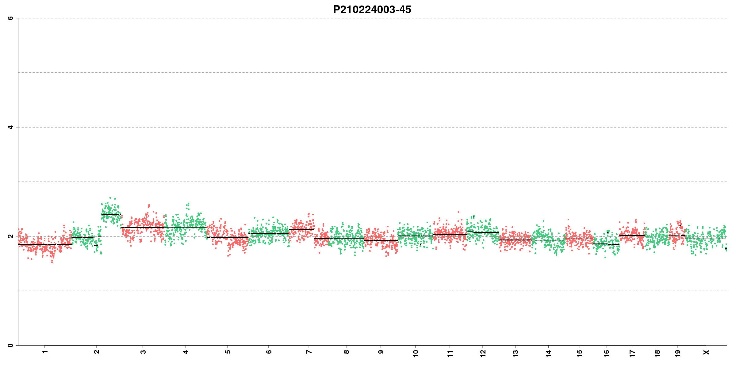


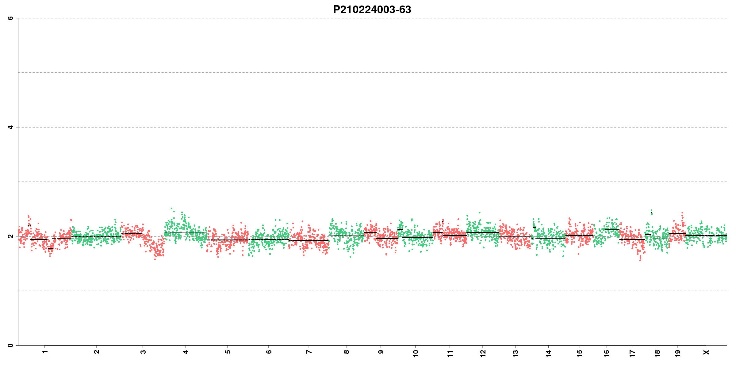

Supplement: Supplementary file 1 [file DataSheet_1.docx]
